# Cell Surface Proteomic Map of HIV Infection Reveals Antagonism of Amino Acid Metabolism by Vpu and Nef

**DOI:** 10.1016/j.chom.2015.09.003

**Published:** 2015-10-14

**Authors:** Nicholas J. Matheson, Jonathan Sumner, Kim Wals, Radu Rapiteanu, Michael P. Weekes, Raphael Vigan, Julia Weinelt, Michael Schindler, Robin Antrobus, Ana S.H. Costa, Christian Frezza, Clary B. Clish, Stuart J.D. Neil, Paul J. Lehner

**Affiliations:** 1 Cambridge Institute for Medical Research, University of Cambridge, Cambridge Biomedical Campus, Cambridge CB2 0XY, UK; 2 Department of Infectious Diseases, King’s College London School of Medicine, Guy’s Hospital, London SE1 9RT, UK; 3 Helmholtz Center Munich, Institute of Virology, 85764 Neuherberg, Germany; 4 Institute of Medical Virology and Epidemiology of Viral Diseases, University Clinic Tübingen, 72076 Tübingen, Germany; 5 MRC Cancer Unit, Hutchison/MRC Research Centre, University of Cambridge, Cambridge Biomedical Campus, Cambridge CB2 0XZ, UK; 6 The Broad Institute of the Massachusetts Institute of Technology and Harvard, Cambridge, MA 02142, USA

## Abstract

Critical cell surface immunoreceptors downregulated during HIV infection have previously been identified using non-systematic, candidate approaches. To gain a comprehensive, unbiased overview of how HIV infection remodels the T cell surface, we took a distinct, systems-level, quantitative proteomic approach. >100 plasma membrane proteins, many without characterized immune functions, were downregulated during HIV infection. Host factors targeted by the viral accessory proteins Vpu or Nef included the amino acid transporter SNAT1 and the serine carriers SERINC3/5. We focused on SNAT1, a β-TrCP-dependent Vpu substrate. SNAT1 antagonism was acquired by Vpu variants from the lineage of SIVcpz/HIV-1 viruses responsible for pandemic AIDS. We found marked SNAT1 induction in activated primary human CD4+ T cells, and used Consumption and Release (CoRe) metabolomics to identify alanine as an endogenous SNAT1 substrate required for T cell mitogenesis. Downregulation of SNAT1 therefore defines a unique paradigm of HIV interference with immunometabolism.

## Highlights

• Unbiased global analysis of T cell surface proteome remodeling during HIV infection
• >100 proteins downregulated, including Nef targets SERINC3/5 and Vpu target SNAT1
• β-TrCP-dependent SNAT1 downregulation acquired by pandemic SIVcpz/HIV-1 viruses
• Uptake of exogenous alanine by SNAT1 critical for primary CD4+ T cell mitogenesis

## Graphical Abstract

**Figure:**
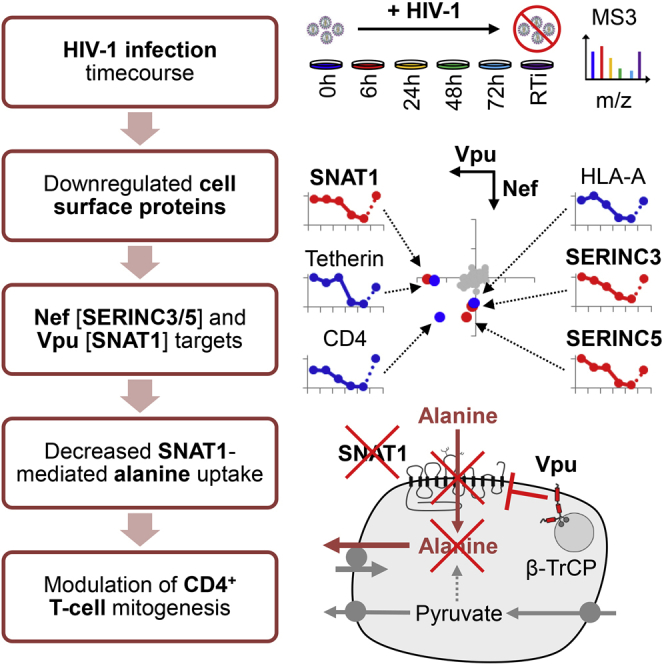

## Teaser

Viruses manipulate host factors to enhance their replication. Matheson et al. use functional proteomics to analyze plasma membrane proteins downregulated during HIV-1 infection. Serine carriers SERINC3/5 and alanine transporter SNAT1 were identified as Nef and Vpu targets, respectively. Antagonism of SNAT1-mediated alanine transport enables viral interference with T cell immunometabolism

## Introduction

HIV-1 viruses of the AIDS pandemic encode four “accessory proteins” (Vif, Vpr, Vpu, and Nef) dispensable for viral replication in vitro, but essential for viral pathogenesis in vivo (Malim and Emerman, 2008). Vpu and Nef are multifunctional adaptors that downregulate cell surface proteins to counteract host-cell restriction and evade the immune response (Haller et al., 2014; Tokarev and Guatelli, 2011). Targets have typically been identified using non-systematic, candidate approaches and include the HIV receptor CD4, the restriction factor tetherin, and the MHC I molecules HLA-A/B (Tokarev and Guatelli, 2011).

Among primate lentiviruses, a correlation is observed between viral pathogenicity and expression of Vpu, with CD4+ T cell decline and progression to AIDS markedly faster in HIV-1 than HIV-2, and increased mortality in chimpanzees infected with SIVcpz (Keele et al., 2009). Vpu induces substrate-specific ubiquitination of CD4 and tetherin through recruitment of the SCF-β-TrCP E3 ligase complex via a constitutively phosphorylated phosphodegron in its cytoplasmic tail (Douglas et al., 2009; Margottin et al., 1998; Mitchell et al., 2009). In the SIV-HIV (SHIV) macaque model of HIV, CD4+ T cell loss is abrogated by deletion of Vpu, scrambling of its transmembrane domain, or mutation of its β-TrCP-binding phosphodegron (Hout et al., 2005; Singh et al., 2003; Stephens et al., 2002). This effect is unlikely to be attributable to loss of Vpu-mediated downregulation of macaque CD4 or tetherin because CD4 is also efficiently downregulated by Nef, and pig-tailed macaque tetherin is antagonized by SIVmac Nef, but not by HIV-1 Vpu (Zhang et al., 2009). Together, these data point to the existence of additional, biologically important, β-TrCP-dependent Vpu substrates.

In this study, we combine plasma membrane enrichment through selective aminooxy-biotinylation (Plasma Membrane Profiling; PMP) with Tandem Mass Tag (TMT) and Stable Isotope Labeling by Amino Acids in Cell Culture (SILAC)-based quantitative proteomics to describe global changes in the cell surface landscape of an HIV-infected T cell, including expression time courses of >800 plasma membrane proteins (Weekes et al., 2013, 2014). Our unbiased, comprehensive analysis reveals downregulation of >100 HIV-1 targets, particularly proteins involved in cell adhesion, leukocyte activation, and transmembrane transport, and is presented as a searchable database to facilitate data mining.

In addition to their known substrates, we show that Vpu is necessary and sufficient for β-TrCP-dependent degradation of the amino acid transporter SNAT1, and Nef is sufficient for downregulation of the serine carriers SERINC3 and SERINC5. We apply an unbiased, CoRe metabolomic approach to identify the non-essential amino acid alanine as an endogenous SNAT1 substrate in primary human CD4+ T cells, and show that extracellular alanine is critical for T cell mitogenesis. Restricting alanine uptake through Vpu-mediated downregulation of SNAT1 therefore represents a viral strategy to regulate immune cell activation.

## Results

### Systematic Time Course Analysis of T Cell Surface Protein Expression during HIV-1 Infection

**Figure 1:**
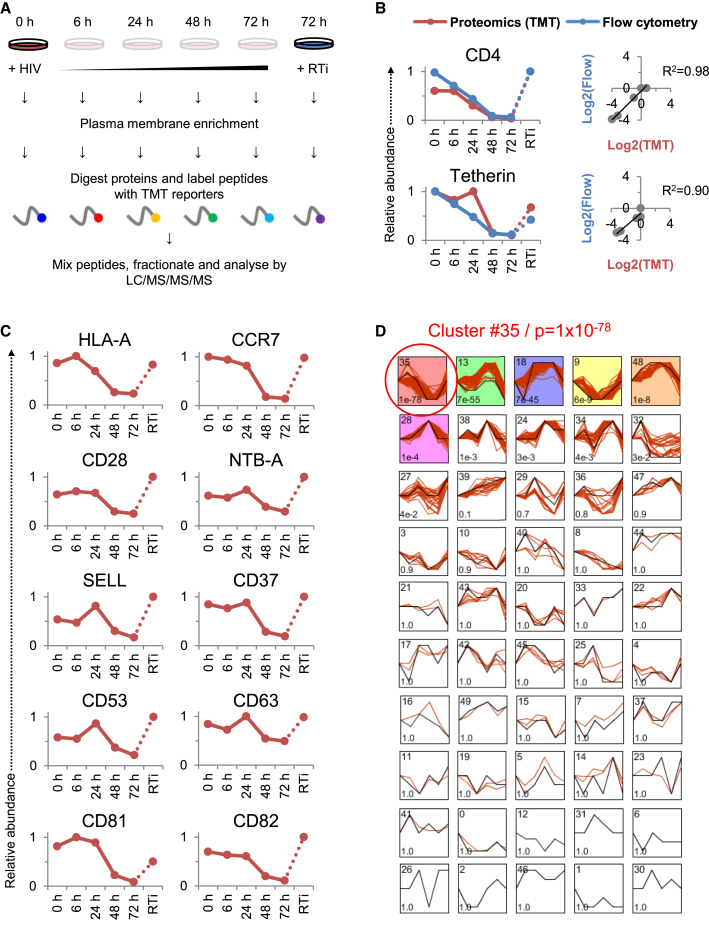
TMT-Based Proteomic Time Course of Plasma Membrane Protein Expression in HIV-1-Infected Cells (A) Workflow of TMT-based 6-plex PMP time course experiment. In subsequent figures, time points 1–5 show plasma membrane protein expression 0, 6, 24, 48, and 72 hr after HIV-1 infection (where 0 hr = uninfected cells), and time point 6 shows plasma membrane protein expression 72 hr after HIV-1 infection in the presence of reverse transcriptase inhibitors (RTi). NL4-3-deltaE-EGFP HIV-1 viruses at an MOI of 10 were used for all proteomic experiments. (B) Comparison of temporal profiles of CD4 and tetherin obtained by proteomic (TMT) versus flow cytometric quantitation. Cells from (A) were stained with anti-CD4 and anti-tetherin antibodies at the indicated time points and analyzed by flow cytometry. Relative abundance is expressed as a fraction of maximum TMT reporter ion or fluorescence intensity. For linear regression, log2(fold change compared with uninfected cells) is shown. (C) Temporal profiles of previously reported targets for HIV-mediated downregulation. (D) Identification of enriched temporal profiles by STEM. Model temporal profiles (black) and matched experimental protein expression profiles (red) are shown. Each box includes a profile identification number (top left) and an unadjusted p value (bottom left). Colored boxes indicate model profiles assigned more proteins than expected by chance alone (Bonferroni-adjusted p values < 0.05). See also Figure S1 and Tables S1 and S2.

To gain a comprehensive, unbiased overview of plasma membrane protein regulation by HIV-1, we used PMP to measure expression levels of cell surface proteins in CEM-T4 T cells infected with HIV-1 (Figure 1A) (Weekes et al., 2013, 2014). By spinoculating cells with Env-deficient, VSVg-pseudotyped virus, we ensured a synchronous, single-round infection, and by using a multiplicity of infection (MOI) sufficient to infect >90% of cells, we minimized confounding effects from bystander (uninfected) cells (Figure S1A). We exploited 6-plex TMT quantitation to compare plasma membrane protein abundance in uninfected cells (0 hr), at 4 time points following HIV-1 infection (6, 24, 48, and 72 hr) and, to control for cellular changes occurring in the absence of de novo viral gene expression, in cells infected for 72 hr in the presence of reverse transcriptase inhibitors (RTi). In total, 2,320 proteins were quantitated, including 804 proteins previously reported to localize to the plasma membrane (Figure S1B). The complete dataset is shown in interactive Table S1, which allows generation of temporal profiles for any quantitated genes of interest.

We observed a strong correlation between expression time courses determined by mass spectrometry and flow cytometry for CD4 (R^2^ = 0.98) and tetherin (R^2^ = 0.90) (Figure 1B), saw marked time-dependent depletion of cell surface HLA-A, and confirmed progressive downregulation of other known HIV-1 targets (CCR7, CD28, NTB-A, SELL, and the tetraspanins CD37/53/63/81/82) (Figure 1C) (Haller et al., 2014; Lambelé et al., 2015; Ramirez et al., 2014; Shah et al., 2010; Swigut et al., 2001; Vassena et al., 2015). Downregulation of CD71 and the chemokine receptors is controversial, with our data suggesting depletion of cell surface CD71, CXCR4, and CCR5 (Figure S1C). As expected, VSVg levels increased immediately after infection, then rapidly declined (Figure S1D).

### Discovery of Cell Surface Targets Depleted by HIV-1

To identify host factors regulated by HIV-1 without observer bias based on known biological function, we used the Short Time Series Expression Miner (STEM) to cluster proteins according to patterns of temporal expression and identify profiles occurring more frequently than expected by chance (Figure 1D). The most enriched profile comprised 134 proteins showing progressive time-dependent downregulation, abolished by reverse transcriptase inhibitors (Cluster #35; p = 10^−78^). Proteins in this cluster, which include CD4, tetherin, and HLA-A, represent candidate HIV-1 cell surface targets (Table S2).

**Figure 2:**
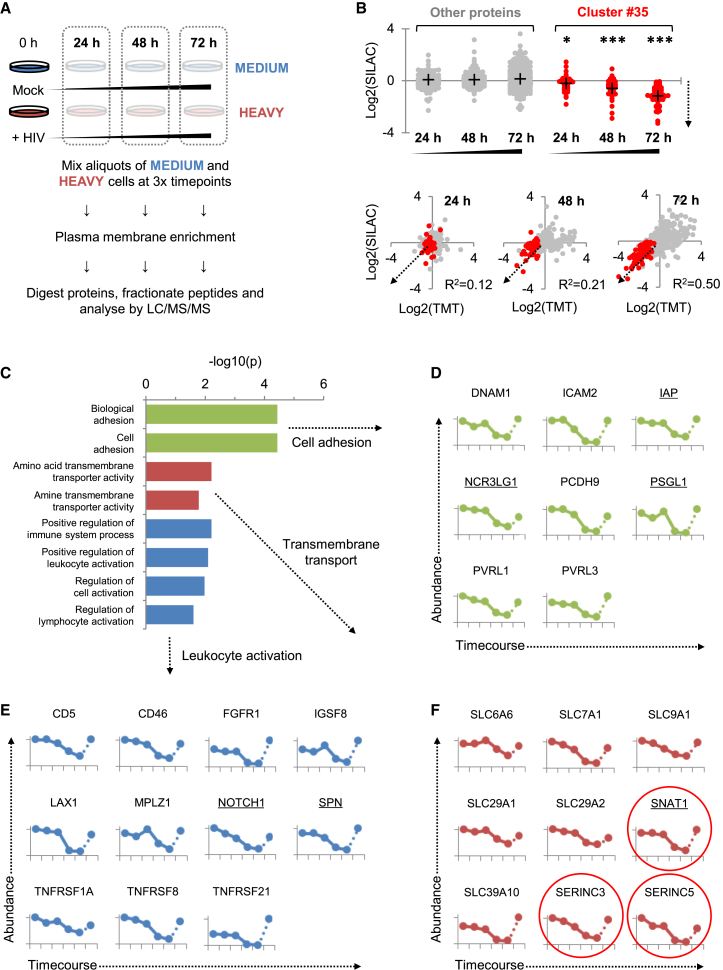
SILAC-Based Proteomic Validation and Functional Analysis of Cell Surface Targets Downregulated by HIV-1 (A) Workflow of SILAC-based 3-way PMP time course experiment. (B) Validation of HIV-1 targets (upper panel) and comparison between SILAC- and TMT-based time course experiments (lower panel). Log2(fold change compared with mock/uninfected cells) at 24, 48, and 72 hr is shown for proteins from Cluster #35 (red) versus all other quantitated proteins (gray). Downregulation by HIV-1 is indicated by dotted arrows. Proteins identified by >1 unique peptide in both TMT and SILAC experiments are shown. Crosses indicate mean values. ^∗^p < 0.05; ^∗∗∗^p < 0.001. (C) Gene ontology “molecular function” and “biological process” terms enriched among proteins from Cluster #35. DAVID functional annotation clusters with adjusted p values < 0.05 and containing terms with Bonferroni-adjusted p values < 0.05 are shown. Further details are included in Table S3. (D–F) Temporal profiles of downregulated proteins associated with cell adhesion (D), leukocyte activation (E), and transmembrane transport (F). Proteomic quantitation and time points are as for Figures 1B–1C. Proteins exhibiting >2-fold downregulation compared with uninfected cells in both TMT and SILAC experiments are shown, and proteins subsequently validated using flow cytometry or immunoblot are underlined. See also Figure S2 and Table S2.

We validated these candidates in an independent infection time course experiment using SILAC as an alternative quantitative proteomic approach (Figures 2A and 2B) and confirmed downregulation of a functionally and structurally diverse set of proteins with available antibody reagents (CD43/47/162 and NOTCH1) by flow cytometry in CEM-T4s and primary human CD4+ T cells infected with HIV-1 (Figure S2A).

### Functional Analysis of Progressively Downregulated HIV-1 Targets

To identify biological functions targeted by HIV-1 in an unbiased fashion, we used the Database for Annotation, Visualization and Integrated Discovery (DAVID) to determine gene ontology “molecular function” and “biological process” annotations over-represented in Cluster #35 compared with other quantitated proteins. The cluster was enriched for terms relating to cell adhesion, leukocyte activation, and transmembrane transport. These categories intersect processes known to be modulated by HIV-1 and provide a framework for interpreting both previously identified and novel HIV-1 targets. We therefore mined our data for downregulated proteins with closely related functions (Figures 2C–2F and Table S3).

Cell adhesion molecules regulate leukocyte trafficking and NK cell killing. Modulation of lymphocyte migration through targeting of SELL and CCR7 (Figure 1C) is proposed to facilitate HIV-1 immune avoidance (Ramirez et al., 2014; Vassena et al., 2015), and downregulation of NTB-A (Figure 1C) protects HIV-infected cells from NK cell lysis (Shah et al., 2010). We now show that HIV-1 also downregulates NCR3LG1 (B7H6; Figure 2D), a ligand for the NK activating receptor NKp30 found on activated monocytic cells in vivo (Brandt et al., 2009; Matta et al., 2013). Flow cytometry confirmed reduced Ig-NKp30 binding to HIV-1-infected CEM-T4 cells (Figure S2B).

HIV-1 replication is critically dependent on the activation state of infected cells, and the virus employs multiple strategies to modulate T cell activation and maximize replication in vivo (Abraham and Fackler, 2012). Attention has focused on downregulation of CD3 by Nef variants of non-pathogenic SIVs, attenuated in HIV-1 Nef (Schindler et al., 2006). Conversely, our data revealed downregulation of numerous other immunoreceptors with important functions in T cell activation (Figure 2E), along with a range of transmembrane transporters with no known roles in the immune system, particularly amino acid transporters (Figure 2F). Since T cell activation requires profound upregulation in amino acid metabolism (Wang et al., 2011), we predict that these proteins have important, but unrecognized, functions in T cell biology.

### Systematic Plasma Membrane Proteomic Analysis of HIV-1 Accessory Proteins Vpu and Nef

**Figure 3:**
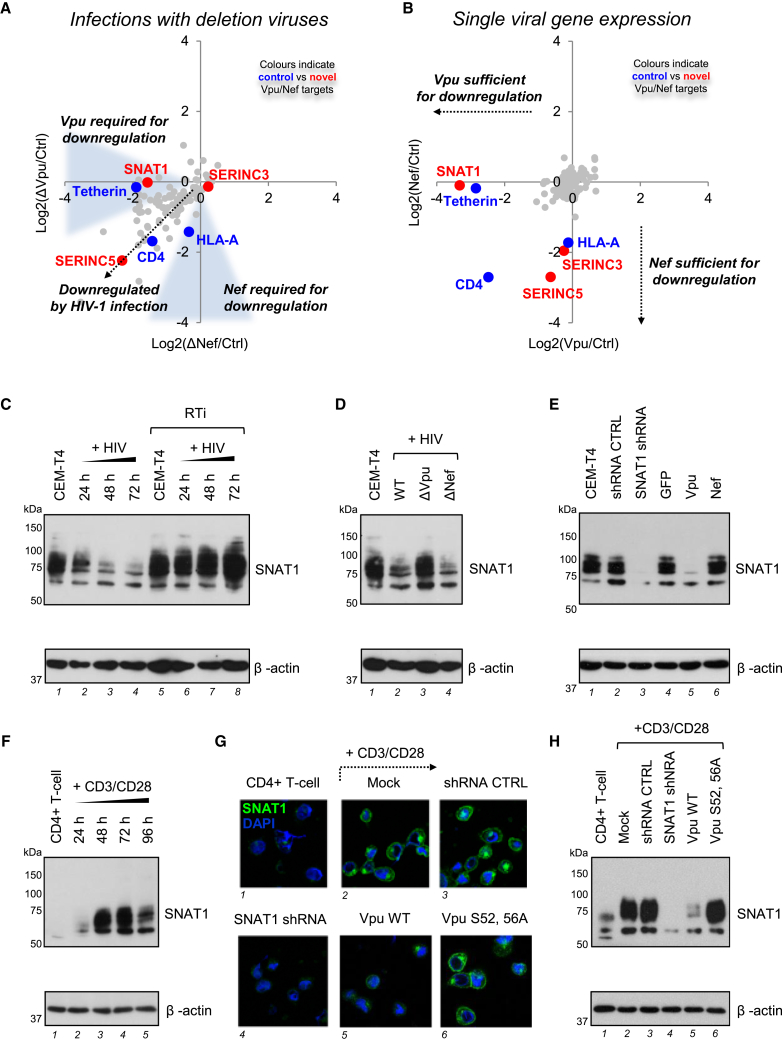
Proteomic Analysis of Vpu and Nef Targets and Identification of SNAT1 as a Vpu Substrate (A and B) SILAC-based quantitation of plasma membrane proteins in cells infected with Vpu-deficient (y axis) versus Nef-deficient (x axis) HIV-1 viruses (A) and cells transduced with Vpu (x axis) versus Nef (y axis) as single genes (B). Log2(fold change compared with uninfected [A] or GFP-transduced [B] cells) is shown for proteins from Cluster #35. Figures S3A and S3B selectively enlarge the lower left quadrant of each scatterplot. Proteins identified by >1 unique peptide are shown. (C) SNAT1 depletion by HIV-1 infection. CEM-T4s infected with WT NL4-3-deltaE-EGFP HIV-1 virus in the presence or absence of RTi were immunoblotted at the indicated time points. An MOI of 10 was used, and infection controls are shown in Figure S4C. (D) Rescue of SNAT1 in the absence of Vpu. CEM-T4s infected with WT, Vpu-deficient, or Nef-deficient HIV-1 NL4-3-deltaE-EGFP viruses were immunoblotted at 48 hr. An MOI of 10 was used, and infection controls are shown in Figure S4C. (E) SNAT1 depletion by Vpu. CEM-T4s stably transduced with GFP, Vpu, or Nef were immunoblotted. Untransduced CEM-T4s and CEM-T4s stably transduced with control or SNAT1-specific shRNAs were included as controls. (F) SNAT1 induction in activated primary T cells. Primary human CD4^+^ T cells activated with CD3/CD28 Dynabeads were immunoblotted at the indicated time points. (G and H) SNAT1 depletion by Vpu in activated primary T cells. Primary human CD4^+^ T cells were activated with CD3/CD28 Dynabeads and mock transduced or transduced with the indicated shRNA or Vpu constructs. After purification by AFMACS (Figure S4F), cells were either rested or re-stimulated with CD3/CD28 Dynabeads and immunoblotted (G) or analyzed by confocal microscopy (H) at 48 hr. See also Figures S3 and S4.

Depletion of most known HIV-1 cell surface targets has been attributed to Vpu and/or Nef (Haller et al., 2014; Tokarev and Guatelli, 2011). To assign downregulation of proteins in Cluster #35 to particular viral genes, we applied an unbiased, systematic approach to define Vpu and Nef substrates. SILAC-based PMP was used to compare expression levels of cell surface proteins in CEM-T4s infected with either Vpu- or Nef-deficient viruses (Figures 3A and S3A), or transduced with Vpu or Nef as single genes (Figures 3B and S3B). As positive controls, we found that Vpu depleted cell surface CD4 and tetherin, while Nef depleted cell surface CD4 and HLA-A (Figures 3A and 3B). Surprisingly, many HIV-1 cell surface targets were downregulated efficiently by both Vpu- and Nef-deficient viruses and not by overexpression of Vpu and Nef as single genes, suggesting Vpu- and Nef-independent mechanisms.

### Targeting of Amino Acid Metabolism by Vpu and Nef

As well as their known targets, we found Vpu to be both necessary and sufficient for downregulation of the amino acid transporter SNAT1 (Figures 2F and 3A–3B), and Nef to be sufficient for downregulation of the serine carriers SERINC3 and SERINC5 (Figures 2F and 3A–3B). This effect was specific within the SERINC family, because SERINC1 was induced rather than downregulated (Figure S1E). While this manuscript was in preparation, SERINC3 and SERINC5 were independently identified as HIV-1 restriction factors using orthogonal approaches (M. Pizzato, personal communication; H. Gottlinger, personal communication). We confirmed restriction of infectious HIV-1 viral production by SERINC5, antagonized by Nef (Figure S3C). Conversely, SNAT1 does not act as an HIV-1 restriction factor (Figure S3D). Instead, we hypothesized that antagonism of SNAT1-dependent amino acid transport by Vpu may modulate T cell activation.

We confirmed Vpu-dependent depletion of endogenous SNAT1 from the plasma membrane of transduced cells by confocal and total internal reflection (TIRF) microscopy (Figures S4A and S4B). As well as decreased expression at the cell surface, depletion of total SNAT1 was seen by immunoblot of CEM-T4s infected with WT or Nef-deficient HIV-1, but not with Vpu-deficient virus (Figures 3C, lanes 2–4, 3D, lanes 2 and 4, and S4C), and by immunoblot of CEM-T4s transduced with Vpu, but not Nef (Figure 3E, lane 5).

Previous studies have suggested that SNAT1 is predominantly expressed in the CNS (Gu et al., 2001; Varoqui et al., 2000). We found SNAT1 protein to be poorly expressed in resting primary human CD4+ T cells but dramatically induced following mitogenic T cell stimulation (Figures 3F, lanes 2–5, and 3G, panel 2). Furthermore, Vpu depletes SNAT1 from activated primary human CD4+ T cells both in the context of viral infection (Figures S4D and S4E) and as a single gene in transduced cells purified by Antibody-Free Magnetic Cell Sorting (AFMACS; Figures 3G, panel 5, 3H, lane 5, and S4F) (Matheson et al., 2014).

### Ubiquitination and β-TrCP-Dependent Endolysosomal Degradation of SNAT1

**Figure 4:**
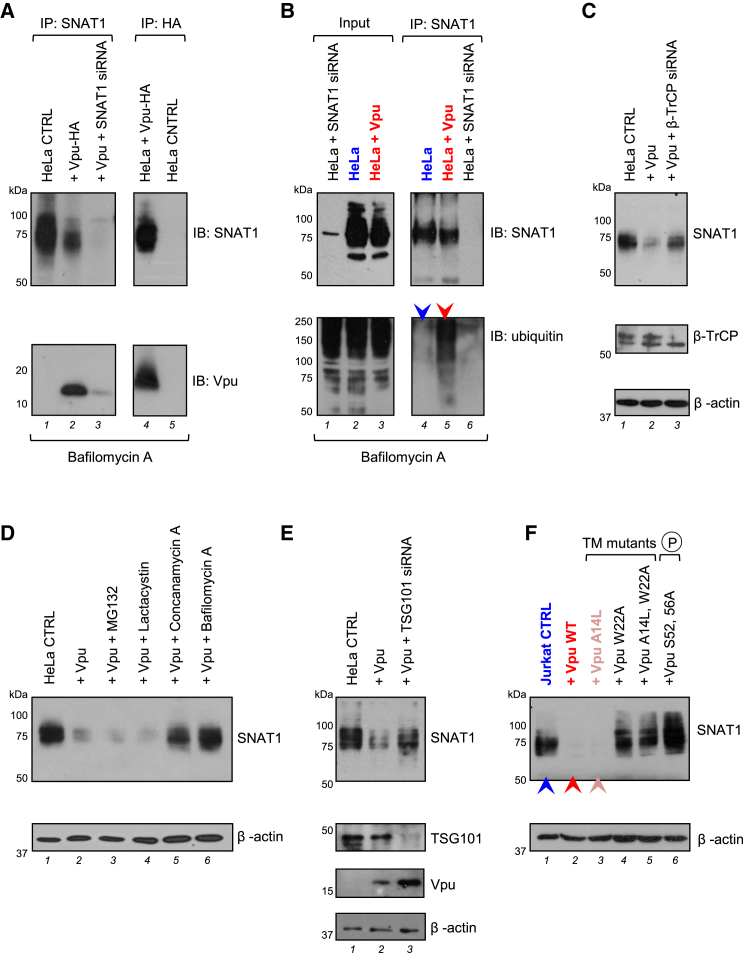
Mechanism of SNAT1 Depletion by Vpu (A) Interaction of SNAT1 with Vpu. HeLa cells stably transduced with Vpu-HA were immunoprecipitated with anti-SNAT1 (G63; first panel) or anti-HA (second panel) antibodies and immunoblotted with anti-SNAT1 (H60) or anti-Vpu antibodies. Untransduced HeLas transfected with SNAT1-specific siRNA were included as controls. (B) Ubiquitination of SNAT1 by Vpu. HeLa cells stably transduced with Vpu-HA were either immunoblotted with anti-SNAT1 (H60) and anti-ubiquitin antibodies (first panel) or immunoprecipitated with anti-SNAT1 (G63) antibody, re-immunoprecipitated with anti-SNAT1 (H60) antibody, and immunoblotted with anti-SNAT1 (H60) and anti-ubiquitin antibodies (second panel). Untransduced HeLas transfected with SNAT1-specific siRNA were included as controls. Ubiquitinated SNAT1 in control (blue arrow) and Vpu-expressing (red arrow) HeLas is highlighted. (C) β-TrCP-dependent depletion of SNAT1. HeLa cells stably transduced with Vpu-HA were transfected with control or β-TrCP-specific siRNA then immunoblotted. (D and E) SNAT1 depletion via an endolysosomal pathway. HeLa cells stably transduced with Vpu-HA were either treated with MG132, lactacystin, concanamycin, or bafilomycin (D) or transfected with control or TSG101-specific siRNA (E) then immunoblotted. (F) Molecular determinants of SNAT1 downregulation. Jurkats stably expressing Vpu WT or indicated Vpu mutants were immunoblotted. Cells transduced with empty vector (blue), Vpu WT (red), and Vpu A14L (pink) are highlighted. The same cells stained with anti-CD4 or anti-tetherin antibodies and analyzed by flow cytometry are shown in Figure S5A. See also Figure S5.

To probe the mechanism of Vpu-mediated SNAT1 depletion, we confirmed that Vpu binds endogenous SNAT1 (Figure 4A, lanes 2 and 4) and leads to SNAT1 ubiquitination (Figure 4B, lane 5). As with CD4 and tetherin, downregulation of SNAT1 is rescued by mutation of the Vpu phosphodegron responsible for β-TrCP recruitment (S52, 56A) (Figures 3G, panel 6, 3H, lane 6, 4F, and S5A) and by RNAi-mediated depletion of β-TrCP (Figure 4C, lane 3).

Vpu mediates degradation of CD4 by hijacking the endoplasmic reticulum-associated degradation (ERAD) pathway (Margottin et al., 1998; Schubert et al., 1998) and antagonizes tetherin by co-opting the endolysosomal degradative pathway (Douglas et al., 2009; Mitchell et al., 2009). SNAT1 degradation is rescued by incubation with vacuolar ATPase inhibitors, but not proteasome inhibitors (Figure 4D, lanes 5 and 6), and by RNAi-mediated depletion of TSG101 (Figure 4E, lane 3), suggesting that, as for tetherin, SNAT1 is degraded in endolysosomes by the ESCRT machinery.

Downregulation of tetherin is abolished by substitution of conserved amino acid residues W22 or A14 in the transmembrane domain of Vpu, and W22 is also critical for downregulation of CD4 (Vigan and Neil, 2010). While the W22A mutation abolished downregulation of all 3 Vpu substrates, SNAT1 and CD4 downregulation were preserved in the presence of the A14L mutation (Figure 4F, lane 4, and Figure S4A). The same pattern of SNAT1 downregulation was observed with equivalent mutations in a patient-derived Vpu (Figure S5B) and with Vpu mutants in the context of viral infection (Figure S5C). The pathway for SNAT1 degradation by Vpu therefore shares the cellular machinery used for antagonism of tetherin but occurs independently of tetherin downregulation and may be dissociated from it by the A14L mutation.

### CoRe Metabolomic Analysis of SNAT1-Depleted Primary Human CD4+ T Cells

When overexpressed in vitro, SNAT1 mediates uptake of a range of small neutral amino acids (Gu et al., 2001; Varoqui et al., 2000). Transport by SNAT1 is Na dependent and sensitive to competition by the model substrate α-methylaminoisobutyric acid (MeAIB), a specific inhibitor of amino acid transport System A (Mackenzie and Erickson, 2004). Based on its functional characteristics and pattern of expression, SNAT1 has primarily been considered a neuronal glutamine transporter (Chaudhry et al., 2002).

**Figure 5:**
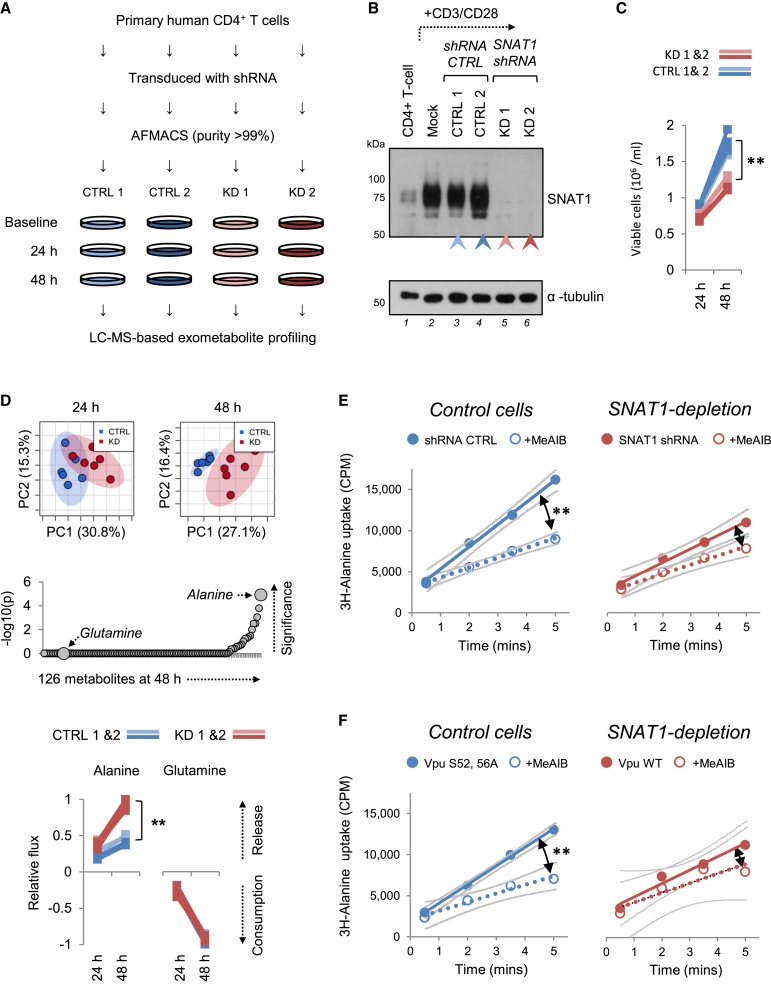
CoRe Metabolomics of Proliferating T Cells and Identification of Alanine Transport by SNAT1 (A) Workflow of CoRe metabolomics experiment. (B) SNAT1 knockdown for CoRe metabolomics experiment. Primary human CD4+ T cells were activated with CD3/CD28 Dynabeads and mock transduced or transduced with the indicated shRNAs. After purification by AFMACS (Figure S6A), cells were either rested or re-stimulated with CD3/CD28 Dynabeads, then immunoblotted at 48 hr. (C) Defective proliferation of SNAT1-depleted primary T cells. Re-stimulated cells from (B) were seeded at equal densities and viable cells enumerated at the indicated time points using CytoCount beads. Data were obtained in triplicate. ^∗∗^p < 0.01. No difference in cell size between the two populations was seen by flow cytometry (Figure S6B). (D) CoRe metabolomic analysis of control and SNAT1-depleted primary T cells. Metabolite compositions of culture supernatants from (C) were determined by LC-MS at baseline, 24, and 48 hr. Data were obtained in triplicate, and Principal component analysis was used to compare net consumption or release of metabolites by control and SNAT1-depleted cells (upper panels). 95% confidence regions are shown. p values for differences in consumption or release of individual metabolites at 48 hr are shown on a negative log scale (middle panel). Net consumption or release of alanine and glutamine is shown scaled to a maximum change of 1 (lower panels). ^∗∗^p < 0.01. (E and F) Impaired alanine uptake by primary T cells depleted of SNAT1 by shRNA (E) or Vpu (F). Cells from Figures 3G–3H were re-stimulated for 48 hr with CD3/CD28 Dynabeads and uptake (counts per minutes; CPM) of 3H-alanine measured at time points from 30 s to 5 min. 3H-alanine transport in the presence of MeAIB is included as a control, and MeAIB-inhibitable uptake is highlighted (black arrows). 95% confidence bands on linear regression lines (indicating rates of uptake) are shown in gray. ^∗∗^p < 0.01. See also Figure S6.

To identify endogenous SNAT1 substrates in primary human CD4+ T cells in an unbiased fashion, we combined an “activation-rest” strategy for shRNA knockdown (Monroe et al., 2014) with Consumption and Release (CoRe) metabolomics (Jain et al., 2012) (Figure 5A). Pure populations of transduced cells expressing control or SNAT1-specific shRNAs were generated by AFMACS (Figures 5B and S6A) (Matheson et al., 2014). After resting for 7–10 days, control and SNAT1-depleted cells were re-stimulated using CD3/CD28 Dynabeads. A marked reduction in proliferation of SNAT1-depleted cells was observed (Figures 5C and S6A). Culture supernatants were sampled at baseline, 24, and 48 hr, and extracellular metabolite fluxes were calculated on a per-cell basis. In total, data for consumption and release of 126 metabolites, including 19 natural amino acids, were used to derive CoRe metabolomic profiles of control and SNAT1-depleted cells. Principal component analysis readily distinguished these profiles, particularly at 48 hr (Figure 5D, upper panels). Surprisingly, across all measured metabolites, the most significant difference was in net alanine release, with no difference in net glutamine consumption (Figure 5D, middle and lower panels).

### Alanine Transport by Endogenous SNAT1 in T Cells

While attention has focused on glutamine, alanine is the paradigmatic substrate for System A amino acid transport (Oxender and Christensen, 1963) and has consistently been found to be a high-affinity substrate for SNAT1 in overexpression studies (Gu et al., 2001; Mackenzie and Erickson, 2004; Varoqui et al., 2000). We therefore hypothesized that the increase in net alanine release caused by SNAT1 depletion may be explained by a decrease in SNAT1-mediated alanine uptake. To test this, 3H-alanine transport was measured directly in AFMACS-purified primary human CD4+ T cells depleted of SNAT1 by expression of a SNAT1-specific shRNA (Figure 5E) or wild-type Vpu (Figure 5F). Whereas alanine uptake in control T cells was markedly reduced by the System A transport inhibitor MeAIB, this effect was abolished in SNAT1-depleted T cells (Figures 5E and 5F), confirming alanine transport by endogenous SNAT1.

### Critical Requirement for Extracellular Alanine in T Cell Mitogenesis

Since alanine is both a non-essential amino acid and excreted by proliferating cells (Jain et al., 2012), including lymphocytes (Figure 5D, lower panels), it appears paradoxical to suggest that a reduction in alanine uptake could result in the mitogenic defect observed in T cells depleted of SNAT1. Nonetheless, we observed a dose-dependent increase in proliferation of CEM-T4 and Jurkat T cells cultured in increasing alanine concentrations (Figure S7A), and a supply of exogenous alanine is required for optimal lymphocyte proliferation in response to PHA (Chuang et al., 1990; Rotter et al., 1979).

**Figure 6:**
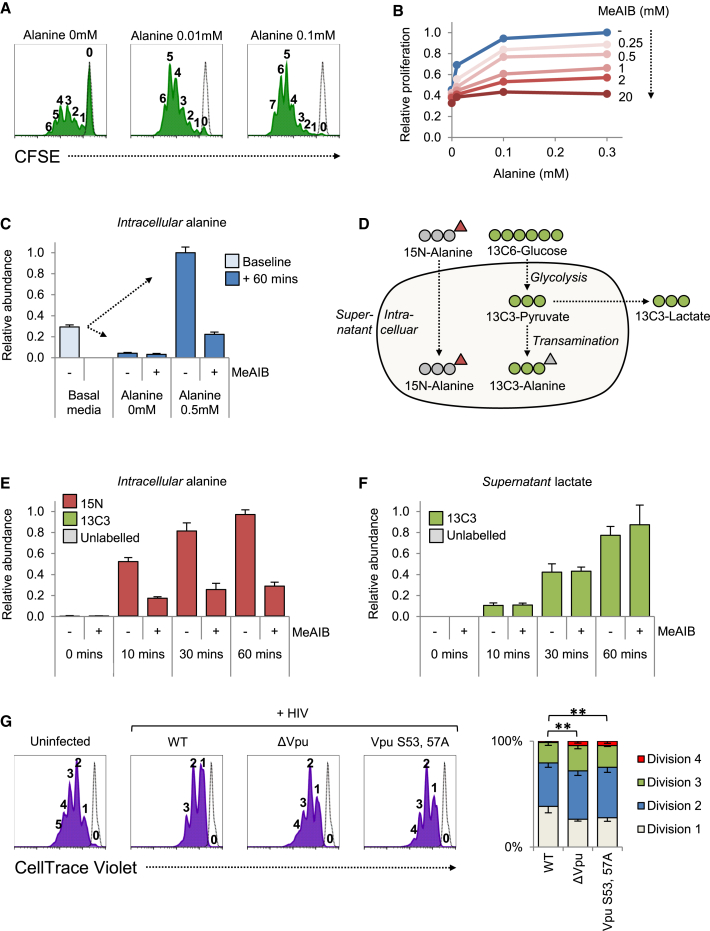
Requirement for Extracellular Alanine in T Cell Mitogenesis (A) Dose-dependent proliferation of primary T cells in response to exogenous alanine. Primary human CD4+ T cells were stained with CFSE, stimulated with CD3/CD28 Dynabeads in media supplemented with alanine at the concentrations indicated, and analyzed by flow cytometry after 120 hr (green filled histograms). Peaks are labeled by division number, and unstimulated cells were included as a control (black dotted lines). Representative data from three independent experiments are shown. (B) Dose-dependent inhibition of primary T cell proliferation by MeAIB. Primary human CD4+ T cells were stimulated with CD3/CD28 Dynabeads in media supplemented with alanine and MeAIB at the concentrations indicated. Viable cells were enumerated using CytoCount beads after 72 hr and numbers expressed as a fraction of the maximum. (C) Regulation of free intracellular alanine pool by System A-dependent alanine uptake. Primary human CD4+ T cells were expanded, rested, and re-stimulated for 48 hr with CD3/CD28 Dynabeads. Cells were then resuspended in media supplemented with alanine at the concentrations indicated in the presence or absence of MeAIB. Abundance of free intracellular alanine at baseline and 60 min is expressed as a fraction of the maximum. Mean values and 95% confidence intervals are shown for data obtained in triplicate. No difference in cell size was observed between 0 and 0.5 mM alanine (Figure S7D, left panel). (D–F) Reconstitution of free intracellular alanine pool by extracellular alanine. Washed cells prepared as in (C) were resuspended in media supplemented with 5.6 mM 13C6-glucose and 0.5 mM 15N-alanine (D) in the presence or absence of MeAIB. Abundances of labeled and unlabeled free intracellular alanine (E) and supernatant lactate (F) at the indicated time points are expressed as a fraction of the maximum. Mean values and 95% confidence intervals are shown for data obtained in triplicate. No difference in cell size was observed in the presence or absence of MeAIB (Figure S7D, right panel). (G) Defective proliferation of primary T cells depleted of SNAT1 by HIV-1. Primary human CD4+ T cells were stained with CellTrace Violet, stimulated with CD3/CD28 Dynabeads, infected with the indicated NL4-3 Vpu 2_87 HIV-1 viruses at an MOI of 3, and analyzed by flow cytometry after 120 hr (violet filled histograms). Peaks are labeled by division number, and unstimulated cells are included as a control (black dotted lines). Representative data for infected (p24+) and uninfected (p24−) cells are shown. Mean percent of infected cells in each generation from four independent experiments are depicted as stacked columns. Error bars indicate SEM. ^∗∗^p < 0.01. See also Figure S7.

We investigated this requirement in primary human CD4+ T cells by activating cells with CD3/CD28 Dynabeads in media supplemented with increasing alanine concentrations. A clear dose response in proliferation from 0 to 0.1 mM was observed (Figure 6A). Furthermore, the effect of increasing alanine concentration was inhibited by MeAIB in a dose-dependent fashion, supporting a role for System A transport in alanine uptake (Figure 6B). Interestingly, exogenous alanine had no effect on the expression of the early T cell activation markers CD69 and CD25 (Figure S7B). The same dissociation of proliferation from early activation has been reported for T cells stimulated in the absence of glutamine (Carr et al., 2010).

### Contribution of Extracellular Alanine to the Free Intracellular Amino Acid Pool

To explain the requirement for exogenous alanine in T cell mitogenesis, we hypothesized that bidirectional transport of alanine at the plasma membrane could result in both uptake of extracellular alanine and net alanine excretion (Figure S7C). We therefore measured the size of the free intracellular alanine pool of primary human CD4+ T cells re-stimulated with CD3/CD28 Dynabeads and resuspended in media either lacking alanine or supplemented with a physiological alanine concentration (Figures 6C and S7D). Intracellular alanine levels were markedly reduced by extracellular alanine depletion, but increased by extracellular alanine supplementation, an effect abolished in the presence of MeAIB. These observations confirm bidirectional flux of alanine across the plasma membrane, resulting in equilibration of intracellular and extracellular alanine concentrations, with alanine uptake mediated by System A transport.

The free intracellular alanine pool may be filled by de novo synthesis through transamination of pyruvate, by release of alanine from proteins by proteasomal or lysosomal degradation, or by uptake of extracellular alanine. To formally distinguish these possibilities, and assess their relative contributions, we resuspended washed cells in media supplemented with physiological levels of heavy isotopologue-labeled 13C6-glucose and 15N-alanine (Figure 6D). The free intracellular alanine pool was rapidly reconstituted by extracellular 15N-alanine, an effect markedly inhibited by MeAIB, with little contribution from unlabeled alanine or alanine generated from 13C-glucose-derived pyruvate (Figures 6E and S7D). Conversely, almost all lactate released from cells was derived from glycolysis of 13C6-glucose (Figures 6F). Finally, MeAIB-inhibitable transamination of 15N-alanine to 15N-glutamate was observed (Figure S7E). Extracellular alanine is therefore rapidly taken up by System A transport in primary human CD4+ T cells and incorporated into the wider cellular metabolite pool.

### Modulation of T Cell Mitogenesis by SNAT1 Downregulation in HIV-1 Infection

As a functional readout for SNAT1 downregulation in the context of viral infection, we examined the effect of Vpu expression on proliferation of primary human CD4+ T cells. Similar to transduction with SNAT1 shRNA, lentiviral delivery of WT Vpu (but not the Vpu S53, 57A phosphodegron mutant) retarded T cell proliferation (Figure S7F). Remarkably, despite antagonism of cell-cycle progression by Vpr (Malim and Emerman, 2008), and Vpu-independent modulation of a range of mitogenic cell surface proteins (Figure 2E), we also observed a significant reduction in proliferation of T cells infected with WT HIV-1, as compared with Vpu-deficient or Vpu S53, 57A phosphodegron mutant viruses (Figure 6G).

### SNAT1 Downregulation by Vpu Variants of Pandemic HIV-1 Viruses

HIV-1 viruses form three main groups, each representing a separate transmission of chimpanzee SIVcpz or gorilla SIVgor to humans: M (or Main, responsible for the AIDS pandemic), O (or Outlier), and N (or New or Non-M, Non-O). Group M viruses are responsible for greater than 90% of all HIV infections and cluster into genetically distinct clades, of which the most widespread are A (East Africa), B (Europe and North America), and C (Southern Africa) (Hemelaar et al., 2011). Among non-human primates, Vpu is found in viruses of the SIVcpz lineage (including HIV-1 and SIVgor), as well as more distantly related guenon monkey viruses (SIVgsn, SIVmus, and SIVmon).

**Figure 7:**
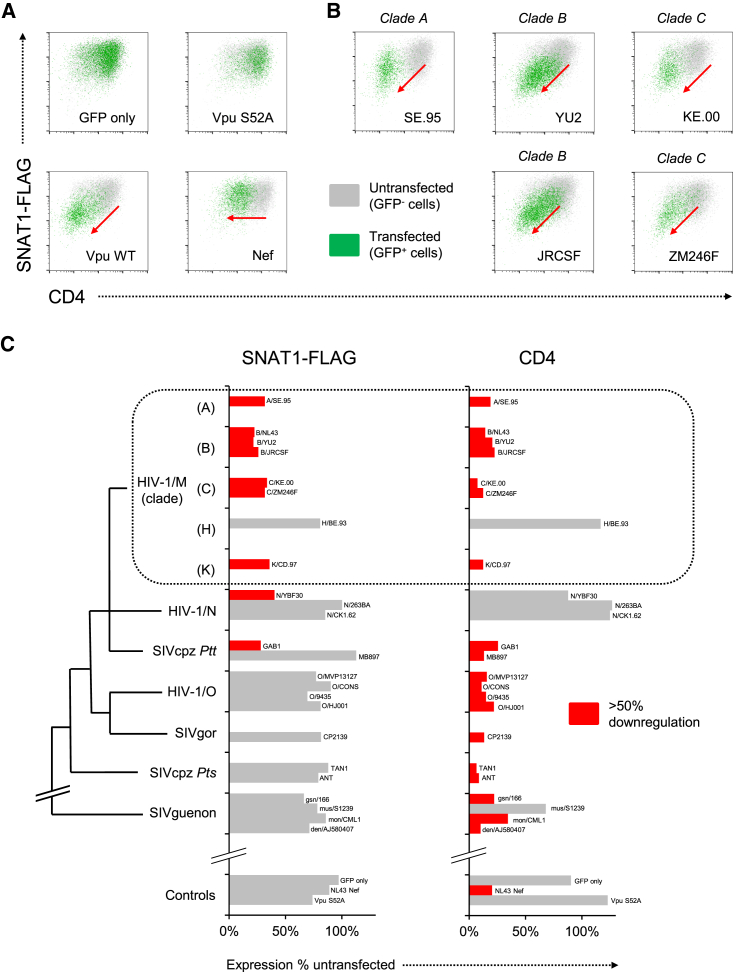
SNAT1 Downregulation by Vpu Variants from Pandemic HIV-1 Viruses (A) Screening strategy for SNAT1 downregulation by naturally occurring Vpu variants. 293Ts stably expressing SNAT1-FLAG and CD4 were transfected with the indicated pCG-IRES-GFP constructs (all based on HIV-1 group M, clade B, strain NL4-3 virus) and analyzed by flow cytometry at 36 hr. Target downregulation is indicated by a shift in the transfected (GFP^+^) cells toward the lower left quadrant (red arrows). (B) SNAT1-FLAG downregulation by Vpu variants from pandemic HIV-1 group M clade A/B/C viruses. As for (A), but cells were transfected with pCG-IRES-GFP constructs encoding Vpu variants from the indicated strains of HIV-1. (C) Phylogenetic analysis of SNAT1-FLAG downregulation by Vpu variants of HIV-1 and SIV viruses. As for (A) and (B), but cells were transfected with pCG-IRES-GFP constructs encoding Vpu variants from the indicated strains of HIV-1 or SIV and downregulation of SNAT1-FLAG or CD4 expressed as ratio of geomean fluorescence intensity between transfected (GFP^+^) and untransfected (GFP^−^) cells. Illustrative phylogenetic relationships are shown, and branch lengths are arbitrary (further details are included in Supplemental Experimental Procedures). HIV-1/M/N/O (HIV-1 group M, N, or O viruses); SIVcpz *Ptt* (SIVs infecting central *P. t. troglodytes* chimpanzees); SIVcpz *Pts* (SIVs infecting eastern *P. t. schweinfruthii* chimpanzees); SIVgor (gorilla SIV); SIVguenon (SIVs infecting guenon monkeys).

To explore the phylogenetic history of Vpu-mediated SNAT1 downregulation, we generated a stable 293T cell line expressing SNAT1-FLAG and CD4 and compared the effects of different Vpu-IRES-GFP constructs. CD4 downregulation is widely conserved and therefore represents a positive control for functional Vpu expression (Sauter et al., 2009). As expected, whereas NL4-3 Vpu (but not Vpu S52A) downregulated both CD4 and SNAT1-FLAG, Nef only downregulated CD4 (Figure 7A). Depletion of cell surface SNAT1-FLAG was conserved across all 6 HIV-1 clade A, B, and C Vpu variants tested (Figures 7A and 7B), but restricted to the SIVcpz *Ptt* lineage giving rise to pandemic HIV-1 group M viruses (Figure 7C). The ability of Vpu to downregulate SNAT1 has therefore been acquired recently and may be critical for the in vivo replication or enhanced pathogenicity of HIV-1 viruses.

## Discussion

In this study, we provide a comprehensive, unbiased temporal map of the cell surface of an HIV-1-infected T cell. Our plasma membrane proteomic approach captures transcriptional and post-transcriptional effects, including protein sequestration and redistribution, and is not limited to known T cell immunoreceptors (Weekes et al., 2014). The study of cell surface proteins downregulated by viruses has uncovered important areas of immunobiology, and manipulation by HIV-1 therefore suggests host factors with unsuspected functions in both viral pathogenesis and cellular physiology. Downregulation is unlikely to reflect a non-specific cellular response to productive viral infection because plasma membrane proteins depleted by HIV-1 exhibit contrasting temporal regulation in cells infected with human cytomegalovirus, even within protein families (Figures S1F and S1G) (Weekes et al., 2014).

Along with CD4 and tetherin, our data identify SNAT1 as the third β-TrCP-dependent Vpu substrate. Other Vpu targets have been proposed, based on candidate approaches: NTB-A, CCR7, CD1d, PVR, SELL, and the tetraspanins CD37/53/63/81/82 (Haller et al., 2014; Lambelé et al., 2015; Matusali et al., 2012; Moll et al., 2010; Ramirez et al., 2014; Shah et al., 2010; Vassena et al., 2015). In general, the mechanisms are not β-TrCP dependent, remain poorly characterized, and may be indirect. Furthermore, the magnitude of downregulation reported has been modest, which may contribute to less-robust phenotypes (Sato et al., 2012). Compared with these targets, our systematic analysis suggests that downregulation of CD4, tetherin, and SNAT1 is qualitatively distinct (Figure S3E), reflecting recruitment of β-TrCP and hijack of enzymatic ubiquitin-mediated degradation. Together with downregulation of CD4, MHC-I, SERINC3, and SERINC5 by Nef, we therefore define a more limited group of highly downregulated HIV-1 accessory protein targets.

Vpu and Nef co-operate in the downregulation of CD4 and tetherin, and loss of function in one gene may be compensated by gain of function in the other (Sauter et al., 2009). The Nef proteins of HIV-2 and most SIVs are able to modulate T cell activation by downregulating CD3 from the surface of infected cells, but Nef has lost this ability in most Vpu-containing viruses. Vpu has therefore been suspected to modulate T cell activation via an alternative pathway (Kirchhoff, 2009). We focused on downregulation of SNAT1 both because it is a direct Vpu target and because the importance of amino acid transport in regulating T cell activation is increasingly recognized (Nakaya et al., 2014; Sinclair et al., 2013). Furthermore, while many transporters are poorly characterized multi-pass transmembrane proteins with few reliable reagents, their plasma membrane location makes them potentially druggable therapeutic targets, and inhibitors already exist for many biochemically defined transport systems.

Induction of SNAT1 mRNA correlates with increased glutamine uptake during activation of murine T cells (Carr et al., 2010), but other candidate glutamine transporters are also induced (Nakaya et al., 2014; Wang et al., 2011), and pre-genomic studies attributed lymphocyte glutamine transport to Systems ASC, L, and N, not System A (Segel, 1992). We therefore used an unbiased systematic approach to identify SNAT1 substrates in primary human CD4+ T cells. Measured differences between alanine fluxes of control and SNAT1-depleted cells could potentially reflect both direct transport effects and secondary effects on synthesis or utilization. Alanine may be synthesized by transamination of pyruvate and glutamine-derived glutamate, and re-analysis of data from a previous CoRe metabolomic screen of NCI-60 cancer cell lines confirmed that release of alanine typically correlates with release of lactate and consumption of glucose and glutamine (Jain et al., 2012). Conversely, we saw no difference between control and SNAT1-depleted cells in release of lactate or consumption of glucose and glutamine (Figures 5D and S6C), suggesting similar rates of alanine synthesis, and confirmed alanine uptake by SNAT1 in primary human CD4+ T cells using a formal transport assay and a concentration of alanine approximating that seen in vivo.

Alanine is the second most abundant amino acid in human plasma, but absent from standard media such as RPMI, and contributed to in vitro cell culture systems by serum supplementation (Rotter et al., 1979). Despite net excretion, we show that the concentration of extracellular alanine dramatically impacts T cell mitogenesis, and uptake of exogenous alanine by System A transport is critical to maintain the free intracellular alanine pool. Alanine is a major constituent of mammalian proteins and may be incorporated into the wider cellular metabolite pool by transamination. In addition, bidirectional transport of alanine at the plasma membrane may be used to drive tertiary active transport of other amino acids (Nicklin et al., 2009). The relative significance of these effects remains to be determined. While amino acid availability is known to regulate immune activation in multiple settings, antagonism of SNAT1 by HIV-1 Vpu is a specific example of viral interference with amino acid immunometabolism.

Modulation of cell surface targets by viruses may enhance viral replication directly, in a cell-autonomous fashion, or indirectly, through effects on non-infected cells or the immune response. It is difficult to account for indirect effects on virus production in vivo using in vitro models. For example, tetherin restricts (Neil et al., 2008) or enhances (Jolly et al., 2010) HIV-1 replication, depending on the assay used. The significance of tetherin as a restriction factor is instead proven by conservation of antagonism across a range of HIV and SIV viruses, and we therefore sought analagous genetic evidence for the importance of SNAT1 in the host-HIV interaction. SNAT1 antagonism was observed for HIV-1 group M Vpu variants from laboratory-adapted viruses and primary patient isolates, including a founder virus strain, X4 and R5 tropic viruses, and related HIV-1 group N and SIVcpz *Ptt* Vpu variants. Remarkably, despite the extraordinary sequence diversity of HIV-1, and the potential to dissociate SNAT1 downregulation from that of CD4 and tetherin, the ability of Vpu to target SNAT1 is therefore conserved across pandemic HIV-1 viruses, suggesting a significant selective advantage. Furthermore, the restriction of SNAT1 downregulation to Vpu variants from the SIVcpz/HIV-1 lineage suggests a specific role in the pathogenesis of these viruses.

## Experimental Procedures

### HIV-1 Infections

For proteomic time course analysis, CEM-T4 T cells were spinoculated with VSVg-pseudotyped NL4-3-dE-EGFP HIV-1 virus at an MOI of 10, aliquots of infected cells harvested sequentially at the indicated time points, and dead cells removed by immunomagnetic depletion prior to PMP.

### Plasma Membrane Enrichment and Peptide Labeling

PMP was performed as previously described (Weekes et al., 2013, 2014) using 2 × 10^7^ viable cells per condition and a “one pot” oxidation and aminooxy-biotinylation reaction to selectively biotinylate plasma membrane glycoproteins before immunoprecipitation with streptavidin beads and on-bead tryptic digestion. For TMT quantitation, cells from each condition were processed separately, and peptide samples were labeled with TMT reagents before pooling. For SILAC quantitation, cells were pre-labeled by propagation in SILAC media and pooled prior to processing together.

### Proteomics and Data Analysis

Peptide samples were fractionated by high-pH reverse-phase high-pressure liquid chromatography (HpRP-HPLC) and analyzed by liquid chromatography coupled to triple-stage (TMT) or tandem (SILAC) mass spectrometry using an Orbitrap Fusion Tribrid (TMT) or Q Exactive (SILAC) mass spectrometer. Reporter ions from TMT-labeled peptides were quantitated from an MS3 scan using Proteome Discoverer. SILAC-labeled peptides were quantitated using MaxQuant.

### Primary Cell Knockdowns

Primary human CD4+ T cells were activated with CD3/CD28 Dynabeads and transduced with lentiviral constructs encoding U6-shRNA knockdown and SFFV-SBP-ΔLNGFR streptavidin-binding affinity tag cassettes. Transduced cells were selected with streptavidin Dynabeads then released by incubation with excess biotin as previously described (AFMACS) (Matheson et al., 2014). Ethical permission for this project was granted by the Cambridgeshire 2 Research Ethics Committee (REC reference 97/092). Informed written consent was obtained from all of the volunteers included in this study prior to providing blood samples.

### CoRe Metabolomics and Data Analysis

AFMACS-purified primary human CD4+ T cells expressing control or SNAT1-specific shRNAs were re-stimulated using CD3/CD28 Dynabeads. After 24 hr, cells were resuspended in 20% conditioned media at equal densities and supernatant samples at baseline, 24, and 48 hr were analyzed by liquid chromatography coupled to mass spectrometry (LC-MS) as previously described (Jain et al., 2012). To account for differential proliferation, viable cells were enumerated at each time point and changes in metabolite concentrations normalized based on average cell numbers.

### 3H-Alanine Uptake

AFMACS-purified primary human CD4+ T cells expressing control or SNAT1-specific shRNAs were re-stimulated using CD3/CD2 min8 Dynabeads. After 48 hr, cells were starved to reduce *trans*-inhibition then resuspended at 37°C in Tyrode’s buffer supplemented with 3H-alanine at a final concentration of 0.5 mM. Aliquots of cells were harvested sequentially over 5 min and uptake terminated by filtering centrifugation through silicone oil before liquid scintillation counting.

### Free Intracellular Amino Acids

Primary human CD4+ T cells were expanded once and then re-stimulated using CD3/CD28 Dynabeads. After 48 hr, cells were resuspended in media supplemented with dialyzed FCS and either unlabeled alanine and glucose or (for stable isotopologue-resolved metabolomics) 15N-alanine and 13C6-glucose at concentrations of 0.5 mM and 5.6 mM, respectively. Aliquots of cells were harvested sequentially over 1 hr, and free intracellular amino acids were extracted from washed cells using dry ice-cold 50% methanol 30% acetonitrile before analysis by LC-MS. Please see Supplemental Experimental Procedures for further details.

## Author Contributions

N.J.M. and P.J.L. conceived the project and wrote the manuscript; N.J.M., J.S., K.W., R.R., M.P.W., R.V., and J.W. performed experiments; M.P.W. and N.J.M. developed proteomic methods; M.S. supplied essential reagents; R.A. conducted proteomic mass spectrometry; A.S.H.C., C.F., and C.B.C. conducted metabolomic mass spectrometry; and S.J.D.N. and P.J.L. supervised the project. J.S., K.W., and R.R. contributed equally to the final manuscript.

## Supplemental Information

Document S1. Supplemental Experimental Procedures and Figures S1–S7

Table S1. Interactive Spreadsheet of All TMT Time Course Data, Related to Figure 1 Interactive spreadsheet enabling generation of temporal profiles of plasma membrane protein expression for any quantitated genes of interest (“Gene search and plots” worksheet). Instructions are included in the spreadsheet. The complete (unfiltered) TMT time course dataset from which plots are generated is included (“Complete TMT timecourse data” worksheet). Protein abundance is depicted on a color scale (red = downregulated; green = upregulated). The numbers of unique peptides, peptides, and peptide spectral matches are shown for each protein, along with ratio counts and variability for each TMT condition.

Table S2. Cluster #35 TMT and SILAC Time Course Data, Related to Figure 1 Spreadsheet of time course data for 134 proteins in Cluster #35 progressively downregulated by HIV infection. Protein abundance is depicted on a color scale (red = downregulated; green = upregulated). As well as data from the TMT proteomic time course experiment, additional data from the SILAC proteomic validation time course experiment are also included. Not all proteins quantitated in the TMT experiment are seen in the SILAC experiment (this is typical for replicate proteomic experiments). The number of unique peptides is shown for each protein (TMT experiment) and each time point (SILAC experiment). Most confidence is attached to proteins identified by >1 unique peptide. Gene ontology cellular component localization information is shown for each protein: PM, plasma membrane; CS, cell surface; XC, extracellular; ShG, short GOCC (a subset of proteins with short membrane-specific GOCC terms but no subcellular assignment; Weekes et al., 2012); Mem, membrane. Where blank, no GOCC annotation is available.

Table S3. Functional Analysis of Proteins in Cluster #35, Related to Figure 2 Spreadsheet of DAVID functional annotation clusters enriched amongst proteins in Cluster #35 progressively downregulated by HIV infection, against a background of all quantitated proteins. Clusters with enrichment scores > 1.3 (equivalent to a geometric means of all included enrichment p values < 0.05) are shown. Fold enrichment, unadjusted and Bonferroni-adjusted p values for individual gene ontology biological process and molecular function terms are shown.

Document S2. Article plus Supplemental Information

## References

[bib1] Abraham L., Fackler O.T. (2012). HIV-1 Nef: a multifaceted modulator of T cell receptor signaling. Cell Commun. Signal..

[bib2] Brandt C.S., Baratin M., Yi E.C., Kennedy J., Gao Z., Fox B., Haldeman B., Ostrander C.D., Kaifu T., Chabannon C. (2009). The B7 family member B7-H6 is a tumor cell ligand for the activating natural killer cell receptor NKp30 in humans. J. Exp. Med..

[bib3] Carr E.L., Kelman A., Wu G.S., Gopaul R., Senkevitch E., Aghvanyan A., Turay A.M., Frauwirth K.A. (2010). Glutamine uptake and metabolism are coordinately regulated by ERK/MAPK during T lymphocyte activation. J. Immunol..

[bib4] Chaudhry F.A., Reimer R.J., Edwards R.H. (2002). The glutamine commute: take the N line and transfer to the A. J. Cell Biol..

[bib5] Chuang J.C., Yu C.L., Wang S.R. (1990). Modulation of human lymphocyte proliferation by amino acids. Clin. Exp. Immunol..

[bib6] Douglas J.L., Viswanathan K., McCarroll M.N., Gustin J.K., Früh K., Moses A.V. (2009). Vpu directs the degradation of the human immunodeficiency virus restriction factor BST-2/Tetherin via a betaTrCP-dependent mechanism. J. Virol..

[bib7] Gu S., Roderick H.L., Camacho P., Jiang J.X. (2001). Characterization of an N-system amino acid transporter expressed in retina and its involvement in glutamine transport. J. Biol. Chem..

[bib8] Haller C., Müller B., Fritz J.V., Lamas-Murua M., Stolp B., Pujol F.M., Keppler O.T., Fackler O.T. (2014). HIV-1 Nef and Vpu are functionally redundant broad-spectrum modulators of cell surface receptors, including tetraspanins. J. Virol..

[bib9] Hemelaar J., Gouws E., Ghys P.D., Osmanov S., WHO-UNAIDS Network for HIV Isolation and Characterisation (2011). Global trends in molecular epidemiology of HIV-1 during 2000-2007. AIDS.

[bib10] Hout D.R., Gomez M.L., Pacyniak E., Gomez L.M., Inbody S.H., Mulcahy E.R., Culley N., Pinson D.M., Powers M.F., Wong S.W., Stephens E.B. (2005). Scrambling of the amino acids within the transmembrane domain of Vpu results in a simian-human immunodeficiency virus (SHIVTM) that is less pathogenic for pig-tailed macaques. Virology.

[bib11] Jain M., Nilsson R., Sharma S., Madhusudhan N., Kitami T., Souza A.L., Kafri R., Kirschner M.W., Clish C.B., Mootha V.K. (2012). Metabolite profiling identifies a key role for glycine in rapid cancer cell proliferation. Science.

[bib12] Jolly C., Booth N.J., Neil S.J. (2010). Cell-cell spread of human immunodeficiency virus type 1 overcomes tetherin/BST-2-mediated restriction in T cells. J. Virol..

[bib13] Keele B.F., Jones J.H., Terio K.A., Estes J.D., Rudicell R.S., Wilson M.L., Li Y., Learn G.H., Beasley T.M., Schumacher-Stankey J. (2009). Increased mortality and AIDS-like immunopathology in wild chimpanzees infected with SIVcpz. Nature.

[bib14] Kirchhoff F. (2009). Is the high virulence of HIV-1 an unfortunate coincidence of primate lentiviral evolution?. Nat. Rev. Microbiol..

[bib15] Lambelé M., Koppensteiner H., Symeonides M., Roy N.H., Chan J., Schindler M., Thali M. (2015). Vpu is the main determinant for tetraspanin downregulation in HIV-1-infected cells. J. Virol..

[bib16] Mackenzie B., Erickson J.D. (2004). Sodium-coupled neutral amino acid (System N/A) transporters of the SLC38 gene family. Pflugers Arch..

[bib17] Malim M.H., Emerman M. (2008). HIV-1 accessory proteins--ensuring viral survival in a hostile environment. Cell Host Microbe.

[bib18] Margottin F., Bour S.P., Durand H., Selig L., Benichou S., Richard V., Thomas D., Strebel K., Benarous R. (1998). A novel human WD protein, h-beta TrCp, that interacts with HIV-1 Vpu connects CD4 to the ER degradation pathway through an F-box motif. Mol. Cell.

[bib19] Matheson N.J., Peden A.A., Lehner P.J. (2014). Antibody-free magnetic cell sorting of genetically modified primary human CD4+ T cells by one-step streptavidin affinity purification. PLoS ONE.

[bib20] Matta J., Baratin M., Chiche L., Forel J.M., Cognet C., Thomas G., Farnarier C., Piperoglou C., Papazian L., Chaussabel D. (2013). Induction of B7-H6, a ligand for the natural killer cell-activating receptor NKp30, in inflammatory conditions. Blood.

[bib21] Matusali G., Potestà M., Santoni A., Cerboni C., Doria M. (2012). The human immunodeficiency virus type 1 Nef and Vpu proteins downregulate the natural killer cell-activating ligand PVR. J. Virol..

[bib22] Mitchell R.S., Katsura C., Skasko M.A., Fitzpatrick K., Lau D., Ruiz A., Stephens E.B., Margottin-Goguet F., Benarous R., Guatelli J.C. (2009). Vpu antagonizes BST-2-mediated restriction of HIV-1 release via beta-TrCP and endo-lysosomal trafficking. PLoS Pathog..

[bib23] Moll M., Andersson S.K., Smed-Sörensen A., Sandberg J.K. (2010). Inhibition of lipid antigen presentation in dendritic cells by HIV-1 Vpu interference with CD1d recycling from endosomal compartments. Blood.

[bib24] Monroe K.M., Yang Z., Johnson J.R., Geng X., Doitsh G., Krogan N.J., Greene W.C. (2014). IFI16 DNA sensor is required for death of lymphoid CD4 T cells abortively infected with HIV. Science.

[bib25] Nakaya M., Xiao Y., Zhou X., Chang J.H., Chang M., Cheng X., Blonska M., Lin X., Sun S.C. (2014). Inflammatory T cell responses rely on amino acid transporter ASCT2 facilitation of glutamine uptake and mTORC1 kinase activation. Immunity.

[bib26] Neil S.J., Zang T., Bieniasz P.D. (2008). Tetherin inhibits retrovirus release and is antagonized by HIV-1 Vpu. Nature.

[bib27] Nicklin P., Bergman P., Zhang B., Triantafellow E., Wang H., Nyfeler B., Yang H., Hild M., Kung C., Wilson C. (2009). Bidirectional transport of amino acids regulates mTOR and autophagy. Cell.

[bib28] Oxender D.L., Christensen H.N. (1963). Evidence for two types of mediation of neutral and amino-acid transport in Ehrlich cells. Nature.

[bib29] Ramirez P.W., Famiglietti M., Sowrirajan B., DePaula-Silva A.B., Rodesch C., Barker E., Bosque A., Planelles V. (2014). Downmodulation of CCR7 by HIV-1 Vpu results in impaired migration and chemotactic signaling within CD4^+^ T cells. Cell Rep..

[bib30] Rotter V., Yakir Y., Trainin N. (1979). Role of L-alanine in the response of human lymphocytes to PHA and Con A. J. Immunol..

[bib31] Sato K., Misawa N., Fukuhara M., Iwami S., An D.S., Ito M., Koyanagi Y. (2012). Vpu augments the initial burst phase of HIV-1 propagation and downregulates BST2 and CD4 in humanized mice. J. Virol..

[bib32] Sauter D., Schindler M., Specht A., Landford W.N., Münch J., Kim K.A., Votteler J., Schubert U., Bibollet-Ruche F., Keele B.F. (2009). Tetherin-driven adaptation of Vpu and Nef function and the evolution of pandemic and nonpandemic HIV-1 strains. Cell Host Microbe.

[bib33] Schindler M., Münch J., Kutsch O., Li H., Santiago M.L., Bibollet-Ruche F., Müller-Trutwin M.C., Novembre F.J., Peeters M., Courgnaud V. (2006). Nef-mediated suppression of T cell activation was lost in a lentiviral lineage that gave rise to HIV-1. Cell.

[bib34] Schubert U., Antón L.C., Bacík I., Cox J.H., Bour S., Bennink J.R., Orlowski M., Strebel K., Yewdell J.W. (1998). CD4 glycoprotein degradation induced by human immunodeficiency virus type 1 Vpu protein requires the function of proteasomes and the ubiquitin-conjugating pathway. J. Virol..

[bib35] Segel G.B., Kilberg M.S., Haussinger D. (1992). Amino Acid Transport in Lymphocytes. Mammalian Amino Acid Transport.

[bib36] Shah A.H., Sowrirajan B., Davis Z.B., Ward J.P., Campbell E.M., Planelles V., Barker E. (2010). Degranulation of natural killer cells following interaction with HIV-1-infected cells is hindered by downmodulation of NTB-A by Vpu. Cell Host Microbe.

[bib37] Sinclair L.V., Rolf J., Emslie E., Shi Y.B., Taylor P.M., Cantrell D.A. (2013). Control of amino-acid transport by antigen receptors coordinates the metabolic reprogramming essential for T cell differentiation. Nat. Immunol..

[bib38] Singh D.K., Griffin D.M., Pacyniak E., Jackson M., Werle M.J., Wisdom B., Sun F., Hout D.R., Pinson D.M., Gunderson R.S. (2003). The presence of the casein kinase II phosphorylation sites of Vpu enhances the CD4(+) T cell loss caused by the simian-human immunodeficiency virus SHIV(KU-lbMC33) in pig-tailed macaques. Virology.

[bib39] Stephens E.B., McCormick C., Pacyniak E., Griffin D., Pinson D.M., Sun F., Nothnick W., Wong S.W., Gunderson R., Berman N.E., Singh D.K. (2002). Deletion of the vpu sequences prior to the env in a simian-human immunodeficiency virus results in enhanced Env precursor synthesis but is less pathogenic for pig-tailed macaques. Virology.

[bib40] Swigut T., Shohdy N., Skowronski J. (2001). Mechanism for down-regulation of CD28 by Nef. EMBO J..

[bib41] Tokarev A., Guatelli J. (2011). Misdirection of membrane trafficking by HIV-1 Vpu and Nef: Keys to viral virulence and persistence. Cell. Logist..

[bib42] Varoqui H., Zhu H., Yao D., Ming H., Erickson J.D. (2000). Cloning and functional identification of a neuronal glutamine transporter. J. Biol. Chem..

[bib43] Vassena L., Giuliani E., Koppensteiner H., Bolduan S., Schindler M., Doria M. (2015). HIV-1 Nef and Vpu Interfere with L-Selectin (CD62L) Cell Surface Expression To Inhibit Adhesion and Signaling in Infected CD4+ T Lymphocytes. J. Virol..

[bib44] Vigan R., Neil S.J. (2010). Determinants of tetherin antagonism in the transmembrane domain of the human immunodeficiency virus type 1 Vpu protein. J. Virol..

[bib45] Wang R., Dillon C.P., Shi L.Z., Milasta S., Carter R., Finkelstein D., McCormick L.L., Fitzgerald P., Chi H., Munger J., Green D.R. (2011). The transcription factor Myc controls metabolic reprogramming upon T lymphocyte activation. Immunity.

[bib46] Weekes M.P., Tan S.Y., Poole E., Talbot S., Antrobus R., Smith D.L., Montag C., Gygi S.P., Sinclair J.H., Lehner P.J. (2013). Latency-associated degradation of the MRP1 drug transporter during latent human cytomegalovirus infection. Science.

[bib47] Weekes M.P., Tomasec P., Huttlin E.L., Fielding C.A., Nusinow D., Stanton R.J., Wang E.C., Aicheler R., Murrell I., Wilkinson G.W. (2014). Quantitative temporal viromics: an approach to investigate host-pathogen interaction. Cell.

[bib48] Zhang F., Wilson S.J., Landford W.C., Virgen B., Gregory D., Johnson M.C., Munch J., Kirchhoff F., Bieniasz P.D., Hatziioannou T. (2009). Nef proteins from simian immunodeficiency viruses are tetherin antagonists. Cell Host Microbe.

