# Supplementary material for: Cell Surface Proteomic Map of HIV Infection Reveals Antagonism of Amino Acid Metabolism by Vpu and Nef
Source: Cell Host Microbe. 2015 Oct 14;18(4):409–23. doi: 10.1016/j.chom.2015.09.003 (PMC4608997; doi:10.1016/j.chom.2015.09.003)
Supplement: Document S1. Supplemental Experimental Procedures and Figures S1–S7 [file mmc1.pdf]

Cell Host & Microbe, Volume *18*

Supplemental Information

Cell Surface Proteomic Map of HIV Infection Reveals Antagonism of Amino Acid Metabolism by Vpu and Nef

Nicholas J. Matheson, Jonathan Sumner, Kim Wals, Radu Rapiteanu, Michael P. Weekes, Raphael Vigan, Julia Weinelt, Michael Schindler, Robin Antrobus, Ana S.H. Costa, Christian Frezza, Clary B. Clish, Stuart J.D. Neil, and Paul J. Lehner

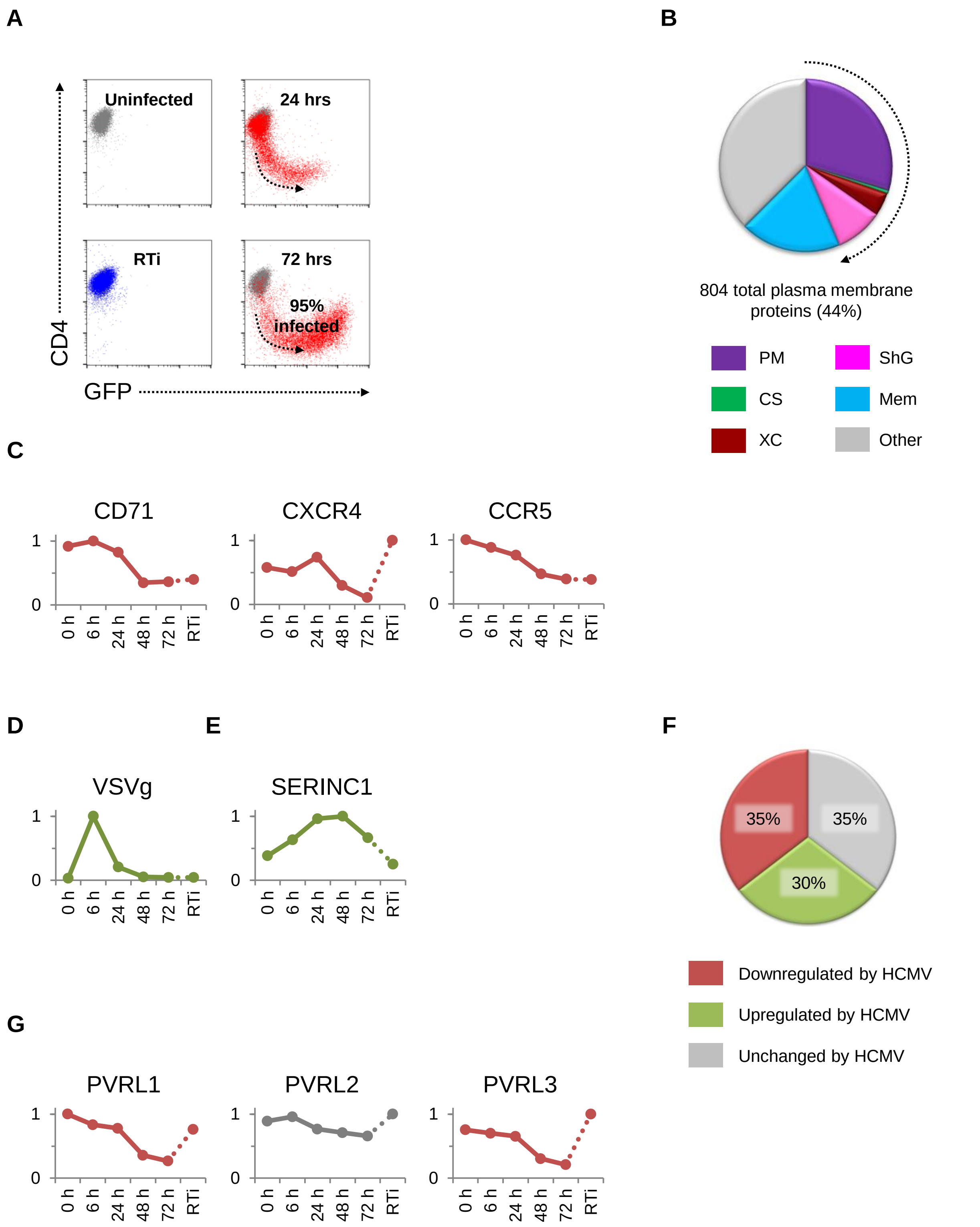

Figure S1. Controls for TMT-based Proteomic Timecourse and Comparison with HCMV Infection, Related to Figure 1

(A) Identification of HIV-infected cells. Cells from **Figure 1A** were stained with anti-CD4 antibody and analysed by flow cytometry. Productive infection with VSVg-pseudotyped NL4-

3-deltaE-EGFP virus results in expression of GFP and downregulation of CD4 by Nef and Vpu. Uninfected cells (grey), cells infected for 24 and 72 hrs (red) and cells infected for 72 hrs in the presence of reverse transcriptase inhibitors (blue) are shown.

(B) Gene Ontology Cellular Compartment (GOCC) annotation of quantitated proteins. PM, plasma membrane; CS, cell surface; XC, extracellular; ShG, short GOCC (a subset of proteins with short membrane-specific GOCC terms but no subcellular assignment (Weekes et al., 2012); Mem, membrane. 1,846 of 2,320 quantitated proteins had GOCC annotations, of which 804 (44%) were indicative of plasma membrane localisation.

(C) Temporal profiles of other reported targets for HIV-mediated downregulation (Drakesmith et al., 2005; Koppensteiner et al., 2014; Michel et al., 2006; Ramirez et al., 2014).

(D) Temporal profile of VSVg.

(E) Temporal profile of SERINC1.

(F) Differential regulation of cell surface proteins by HIV and HCMV infection. 79 proteins from Cluster #35 (cell surface proteins progressively downregulated by HIV) were previously quantitated in HCMV-infected fibroblasts (Weekes et al., 2014) and found to be variably upregulated, downregulated, or unchanged by HCMV infection.

(G) Temporal profiles of nectins (PVRL1-3) revealing differential regulation by HIV and HCMV. Nectin-2 (PVRL2, CD112) is targeted for proteasomal degradation by co-operation between the HCMV proteins UL141 and US2. Conversely, in HIV-1-infected cells (as shown), we observed downregulation of nectin-1 (PVRL1, CD111) and nectin-3 (PVRL3, CD113), but not nectin-2.

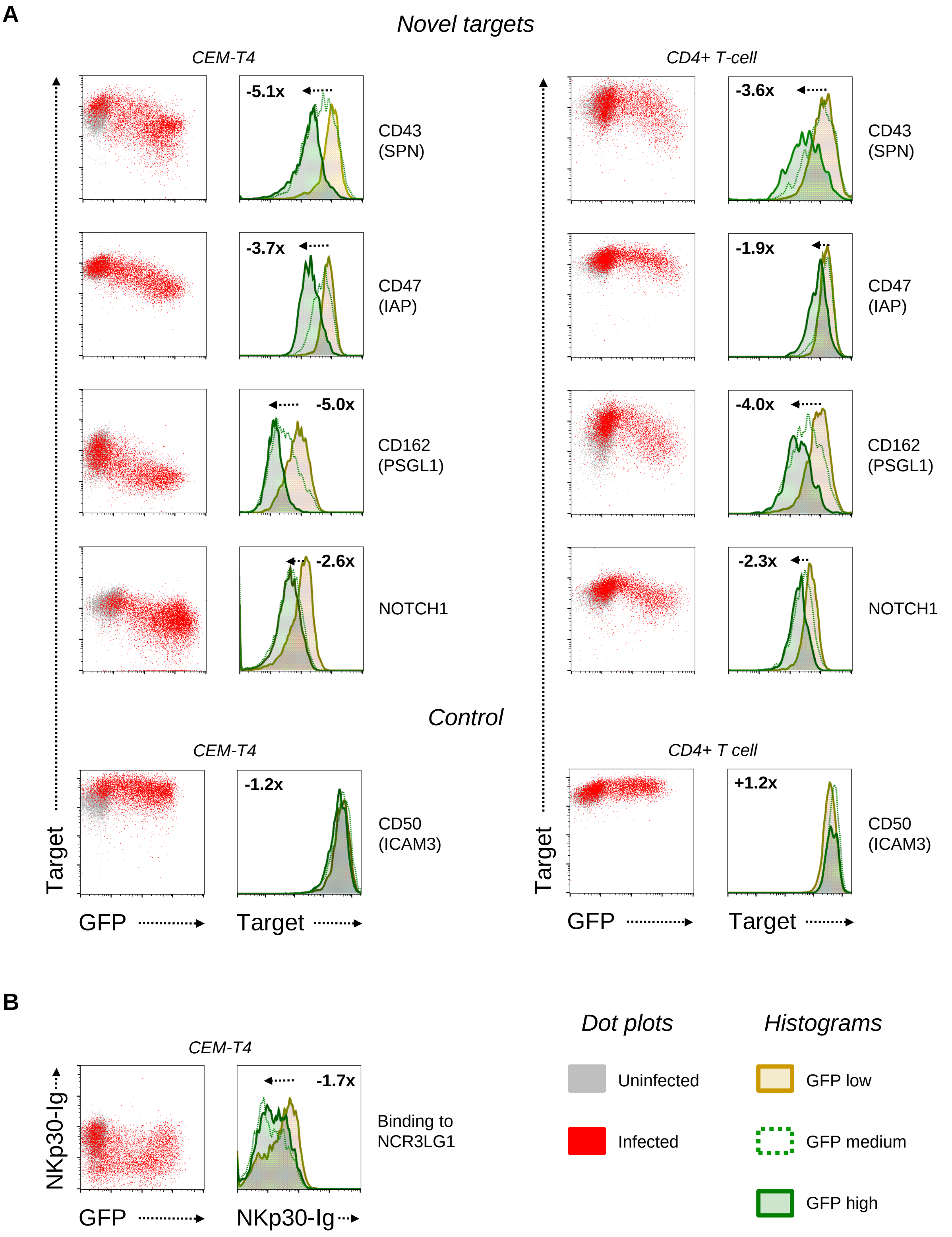

Figure S2. Flow Cytometric Validation of Novel HIV-1 Targets, Related to Figure 2

(A) Validation of novel HIV-1 targets by flow cytometry in CEM-T4s and primary T-cells. CEM-T4s or primary human CD4+ T-cells stimulated with CD3/CD28 Dynabeads were stained with antibodies against the indicated targets 48 hrs after infection with NL4-3-deltaE-

EGFP HIV-1 virus typically at an MOI of 1. Target expression is shown for GFP low, medium and high cells and fold-change in geomean fluorescence intensity high/low is indicated. Negative values denote downregulation in productively infected (GFP-expressing) cells. CD50 abundance was unchanged by HIV-infection in the TMT dataset and is included as a control.

(B) Downregulation of NCR3LG1. CEM-T4s were stained with NKp30-Ig fusion protein (endogenous NCR3LG1 ligand) 48 hrs after infection with NL4-3-deltaE-EGFP HIV-1 virus at an MOI of 1. As in (A) NKp30-Ig binding is shown for GFP low, medium and high cells and fold-change high/low is indicated.

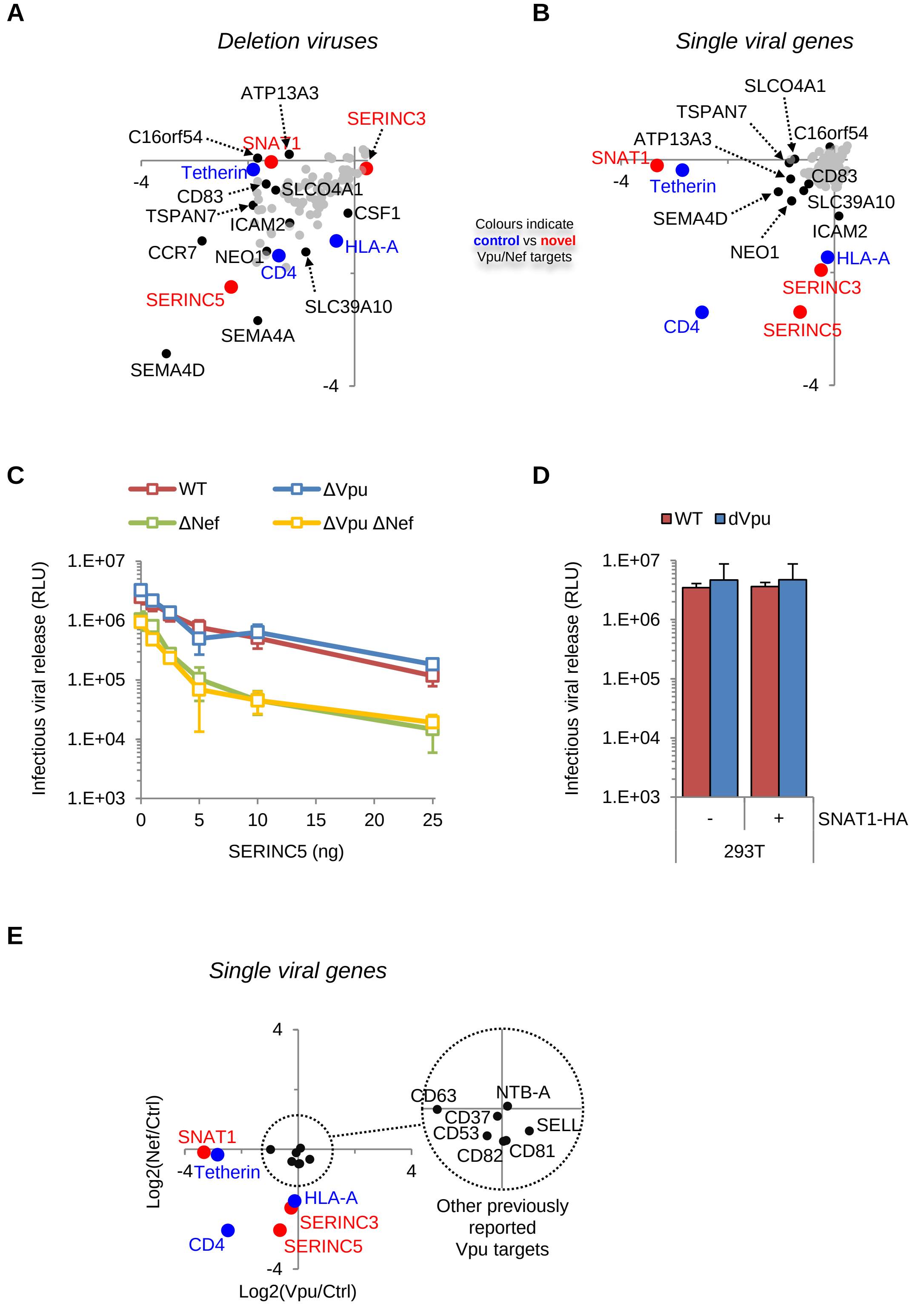

Figure S3. Novel Vpu and Nef targets, Related to Figure 3

(A-B) SILAC-based quantitation of plasma membrane proteins in cells infected with Vpu-deficient (y-axis) versus Nef-deficient (x-axis) viruses (A; enlarged left lower quadrant of scatterplot shown in **Figure 3A**) or transduced with Vpu (x-axis) versus Nef (y-axis) as single

genes (B; enlarged left lower quadrant of scatterplot shown in **Figure 3B**). Outliers of potential interest are labelled.

(C) Restriction of HIV-1 virus production by SERINC5. 293Ts were co-transfected with the indicated pNL4-3 HIV-1 molecular clones plus increasing concentrations of pCR3.1-SERINC5 and 48 hr culture supernatants assayed for infectious viral release using HeLa-TZM-bl cells. Mean values and 95% confidence intervals are shown.

(D) No effect on HIV-1 virus production by SNAT1. Control and SNAT1-HA-expressing 293Ts were infected with the indicated VSVg-pseudotyped NL4-3 HIV-1 viruses at an MOI of 2 and 48 hr culture supernatants assayed for infectious virus release using HeLa-TZM-bl cells. Mean values and 95% confidence intervals are shown.

(E) Comparison of SNAT1 with known Vpu targets. As for (B) and Figure 3B, but showing downregulation of SNAT1, CD4 and tetherin compared with previously reported Vpu targets NTB-A, SELL, CD37, CD53, CD63, CD81 and CD82. HLA-A (downregulated by Nef) is also shown. CCR7, CD1d and PVR (CD155) were not quantitated in this experiment, but CCR7 was downregulated by both ΔVpu and ΔNef viruses. CD53 was quantitated on the basis of a single PSM.

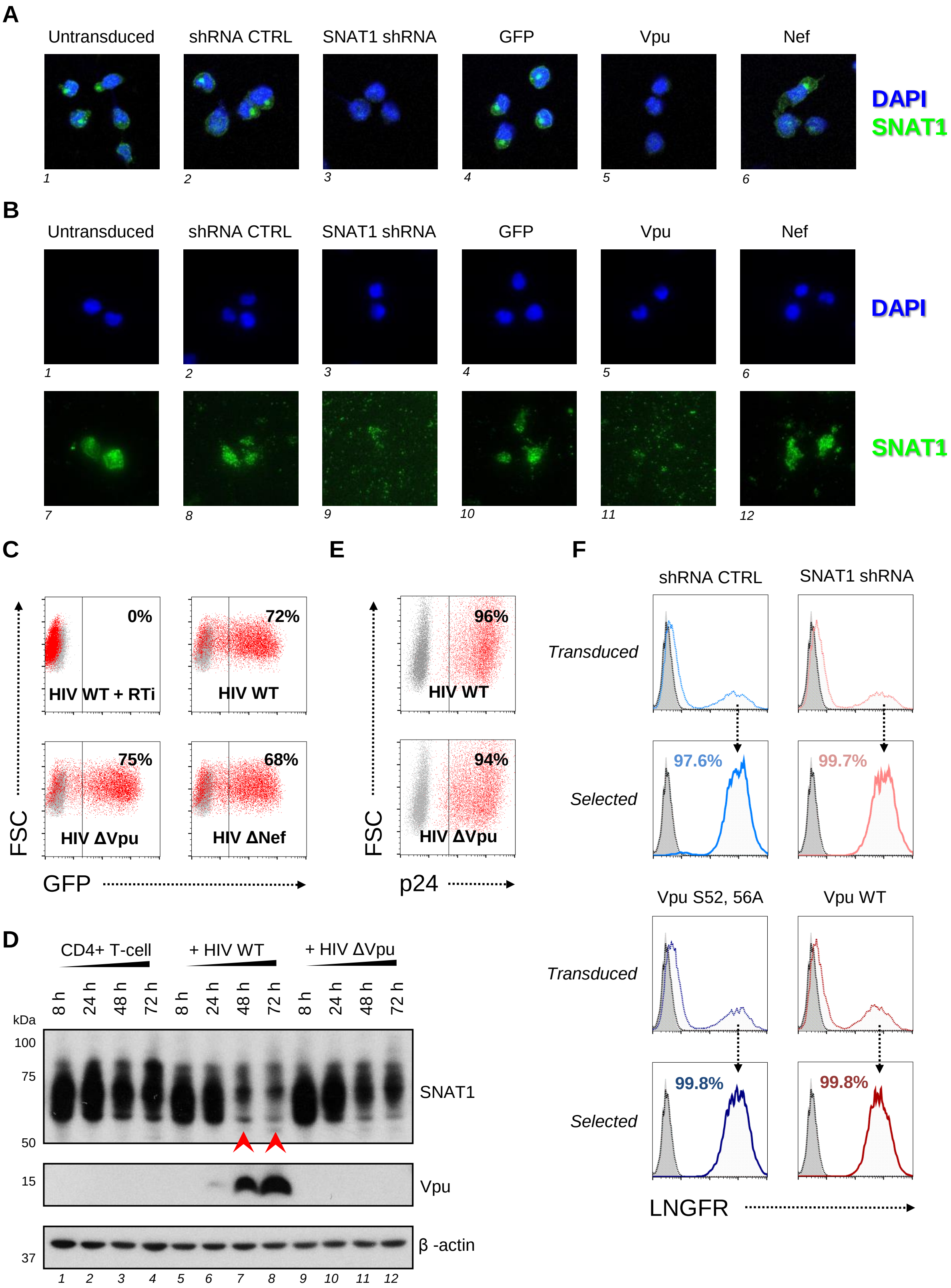

Figure S4. Validation of SNAT1 Depletion, Related to Figure 3

(A-B) Cell surface SNAT1 depletion by Vpu. Cells from **Figure 3E** were stained with anti-SNAT1 antibody (green) and DAPI (blue) and analysed by confocal microscopy (A) or TIRF

microscopy (B). TIRF microscopy enables selective visualisation of the plasma membrane (SNAT1; lower panels) with widefield images (DAPI; upper panels) included as controls.

(C) Infection controls for **Figures 3C-D**. Cells from **Figures 3C-D** were analysed by flow cytometry 48 hrs after infection with the indicated NL4-3-deltaE-EGFP HIV-1 viruses. In each case % productively infected (GFP+) cells is indicated. Uninfected cells (grey) are also shown.

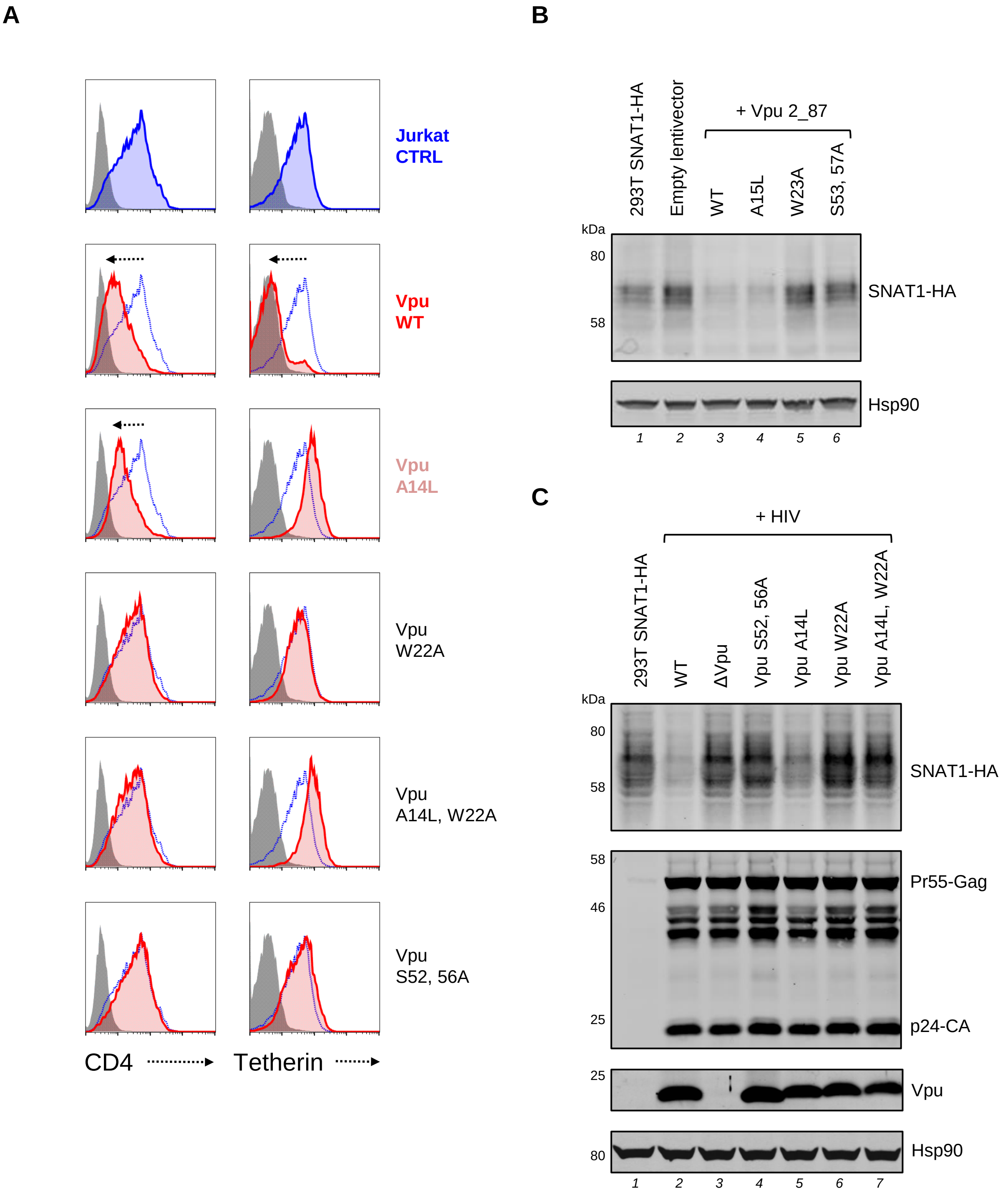

Figure S5. Controls for Mechanism of SNAT1 Depletion, Related to Figure 4

(A) Molecular determinants of CD4 and tetherin downregulation. Cells from **Figure 4F** were stained with anti-CD4 or anti-tetherin antibodies and analysed by flow cytometry. Jurkats transduced with empty vector (blue) or Vpu variants (red) are shown. Unstained cells (CD4) or cells stained with secondary antibody only (tetherin) are included as controls (grey).

(B) Depletion of SNAT1 by Vpu 2_87. 293Ts stably expressing SNAT1-HA were transduced with the indicated Vpu constructs at an MOI of 2 then immunoblotted with anti-HA and HSP90 (loading control) antibodies.

(C) Molecular determinants of SNAT1 depletion by HIV-1. 293Ts stably expressing SNAT1-HA were infected with the indicated VSVg-pseudotyped NL4-3 HIV-1 viruses at an MOI of 2 then immunoblotted with anti-HA, anti-p24, anti-Vpu and anti-HSP90 (loading control) antibodies.

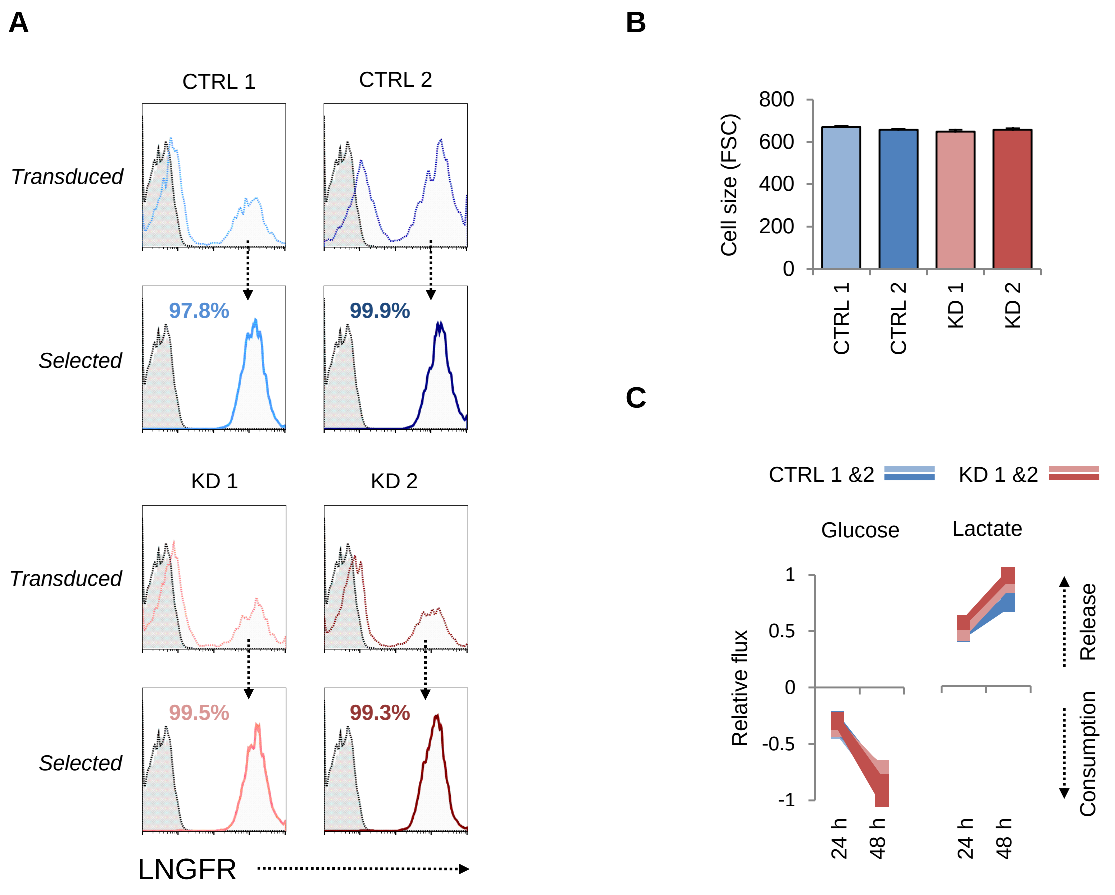

Figure S6. Controls for CoRe Metabolomics, Related to Figure 5

(A) Enrichment of transduced cells by AFMACS. Transduced cells from **Figures 5A-D** were stained with anti-LNGFR antibody before and after selection by AFMACS (red and blue lines). In each case, unstained cells (grey) and control cells stained with anti-LNGFR antibody (dotted line) are included and % LNGFR positive cells after selection are shown.

(B) No change in size of T-cells in response to SNAT1 depletion. Cells from **Figure 5B** were analysed by flow cytometry 48 hrs after re-stimulation with CD3/CD28 Dynabeads. Cell size is indicated by intensity of forward-scattered light (FSC). Mean values and 95% confidence intervals are shown for data obtained in triplicate.

(C) No change in glucose uptake or lactate release by SNAT1 depleted T-cells. As for **Figure 5D**, but net consumption or release of glucose and lactate by control and SNAT1-depleted cells is shown, scaled to a maximum change of 1.

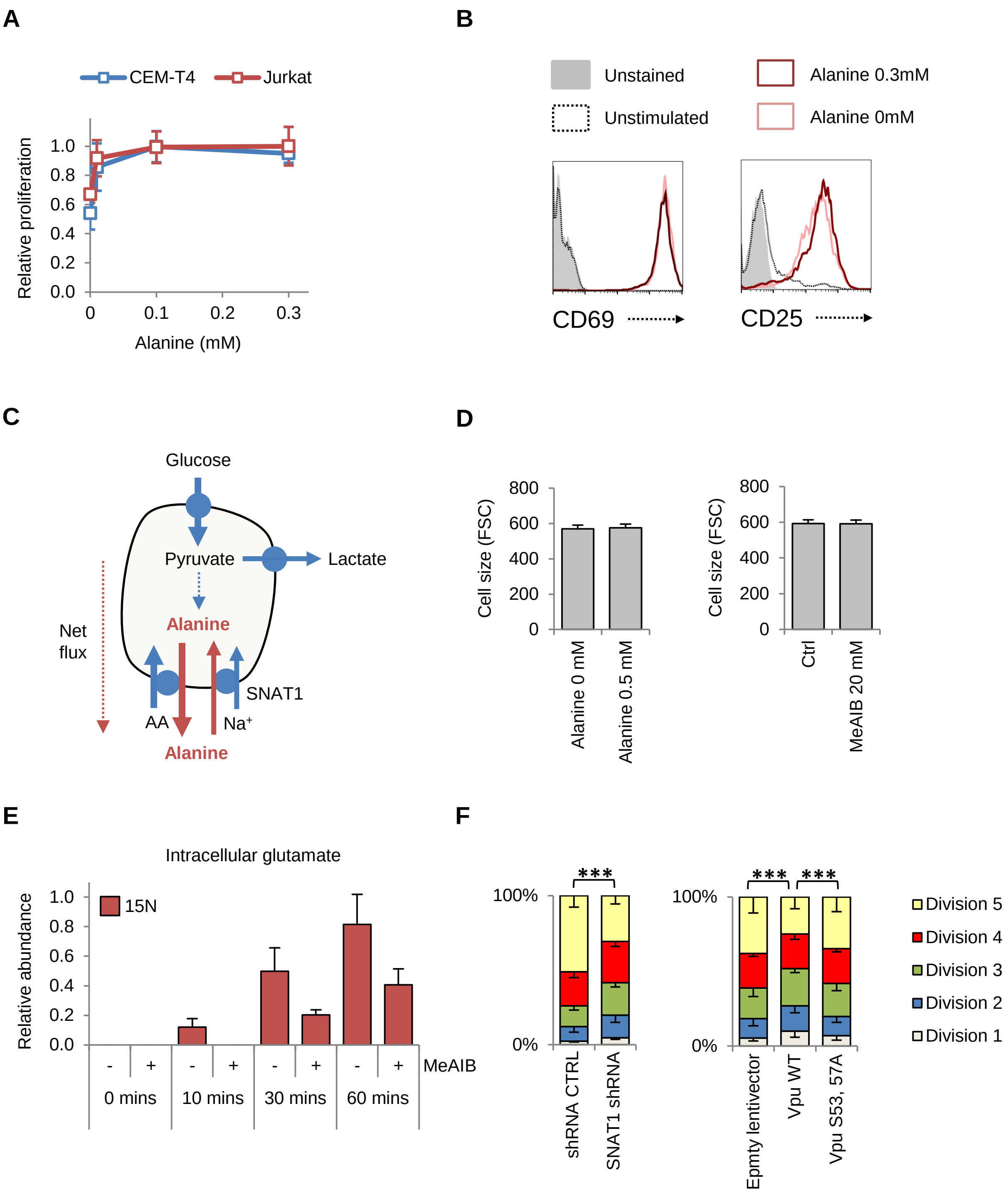

Figure S7. Controls for Free Intracellular Amino Acids, Related to Figure 6

(A) Dose-dependent proliferation of CEM-T4s and Jurkats in response to exogenous alanine. CEM-T4 and Jurkat T-cells were seeded in phenol red-free media supplemented with alanine at the concentrations indicated. Viable cells were enumerated using an MTT assay after 72 hrs and optical density expressed as fraction of maximum.

(B) Preserved CD69 and CD25 expression in the absence of alanine. As for **Figure 6A** but cells were stained with conjugated antibodies against CD69 and CD25 and analysed by flow cytometry after 24 hrs.

SUPPLEMENTAL TABLES

Please see supplied Excel spreadsheets for **Tables S1-3**.

Table S1. Interactive Spreadsheet of All TMT Timecourse Data, Related to Figure 1

Interactive spreadsheet enabling generation of temporal profiles of plasma membrane protein expression for any quantitated genes of interest ("Gene search and plots" worksheet). Instructions are included in the spreadsheet. The complete (unfiltered) TMT timecourse dataset from which plots are generated is included ("Complete TMT timecourse data" worksheet). Protein abundance is depicted on a colour scale (red = downregulated; green = upregulated). The number of unique peptides, peptides and peptide spectral matches are shown for each protein, along with ratio counts and variability for each TMT condition.

Table S2. Cluster #35 TMT and SILAC Timecourse Data, related to Figure 1

Spreadsheet of timecourse data for 134 proteins in Cluster #35 progressively downregulated by HIV infection. Protein abundance is depicted on a colour scale (red = downregulated; green = upregulated). As well as data from the TMT proteomic timecourse experiment, additional data from the SILAC proteomic validation timecourse experiment are also included. Not all proteins quantitated in the TMT experiment are seen in the SILAC experiment (this is typical for replicate proteomic experiments). The number of unique peptides is shown for each protein (TMT experiment) and each timepoint (SILAC experiment). Most confidence is attached to proteins identified by >1 unique peptide. Gene Ontology Cellular Component localisation information is shown for each protein: PM, plasma membrane; CS, cell surface; XC, extracellular; ShG, short GOCC (a subset of proteins with short membrane-specific GOCC terms but no subcellular assignment (Weekes et al., 2012); Mem, membrane. Where blank, no GOCC annotation is available.

Table S3. Functional Analysis of Proteins in Cluster #35, Related to Figure 2

Spreadsheet of DAVID functional annotation clusters enriched amongst proteins in Cluster #35 progressively downregulated by HIV infection, against a background of all quantitated proteins. Clusters with enrichment scores >1.3 (equivalent to a geometric means of all included enrichment p-values <0.05) are shown. Fold enrichment, unadjusted and Bonferroni-adjusted p-values for individual Gene Ontology Biological Process and Molecular Function terms are shown.

EXTENDED EXPERIMENTAL PROCEDURES

General Cell Culture

CEM-T4s (AIDS Reagent Program, Division of AIDS, NIAD, NIH: Dr J.P. Jacobs) (Foley et al., 1965) and Jurkats (Neil laboratory stocks) were cultured in RPMI supplemented with 10% FCS, 100units/ml penicillin and 0.1mg/ml streptomycin at 37°C in 5% CO_2. HEK 293Ts and HeLas (Lehner laboratory stocks) were cultured in DMEM supplemented with 10% FCS, 100units/ml penicillin and 0.1mg/ml streptomycin at 37°C in 5% CO_2. All cells were confirmed to be mycoplasma negative (Lonza MycoAlert).

Stable Isotope Labelling with Amino Acids in Cell Culture (SILAC)

For SILAC labelling, CEM-T4s were grown for at least 7 cell divisions in SILAC RPMI lacking lysine and arginine (Thermo Scientific) supplemented with 10% dialysed FCS (Gibco), 100units/ml penicillin and 0.1mg/ml streptomycin, 280mg/L proline (Sigma) and light (K0, R0; Sigma), medium (K4, R6; Cambridge Isotope Laboratories) or heavy (K8, R10; Cambridge Isotope Laboratories) $^{13}C/^{15}N$-containing lysine (K) and arginine (R) at 50mg/L.

Defined Culture Media

For experiments requiring stable isotope-labelled or defined concentrations of alanine plus/minus glucose, RPMI lacking amino acids and glucose (USBio) was supplemented with 10% dialysed FCS (Gibco), 50x RMPI amino acids, 10mM HEPES and alanine, glucose and glutamine at the concentrations indicated (all Sigma unless otherwise stated).

Primary Cell Isolation and Culture

Primary human CD4+ T cells were typically isolated from peripheral blood by density gradient centrifugation through Lympholyte-H (Cedarlane Laboratories) and negative selection using the Dynabeads Untouched Human CD4 T Cells kit (Invitrogen) according to the manufacturer's instructions. Purity was assessed by flow cytometry for CD3 and CD4 and routinely found to be ≥95%. Cells were cultured in RPMI supplemented with 10% FCS, 30U/ml recombinant human IL-2 (PeproTech), 100units/ml penicillin and 0.1mg/ml streptomycin at 37°C in 5% CO_2 and activated within 48hrs using Dynabeads Human T-Activator CD3/CD28 beads (CD3/CD28 Dynabeads; Invitrogen) according to the manufacturer's instructions.

For primary activation, cells were typically resuspended with CD3/CD28 Dynabeads at a density of 1-2x10^6 cells/ml in flat-bottomed plates or flasks with a 1:1 bead:cell ratio. Cell densities and culture areas were scaled in proportion. Cultures were expanded every 2-3

days using fresh media (including IL-2) and aiming to maintain cell density ≤2x10^6 cells/ml. For secondary activation, CD3/CD28 Dynabeads were removed after 7-10 days using a DynaMag-2 magnet (Invitrogen), and cells washed and resuspended in fresh media before re-stimulation according to the same protocol.

Ethics Statement

Ethical permission for this project was granted by the Cambridgeshire 2 Research Ethics Committee (REC reference 97/092). Informed written consent was obtained from all of the volunteers included in this study prior to providing blood samples.

HIV-1 Molecular Clones

pNL4-3-deltaE-EGFP (derived from the HIV-1 molecular clone pNL4-3 but encoding Enhanced Green Fluorescent Protein (EGFP) in the *env* open reading frame, rendering Env non-functional) was obtained through the AIDS Reagent Program, Division of AIDS, NIAD, NIH: Drs Haili Zhang, Yan Zhou, and Robert Siliciano (Zhang et al., 2004) and the complete sequence verified by Sanger sequencing (Source BioScience). To generate Vpu- and Nef-deficient viruses an XhoI-EcoRI restriction fragment was subcloned into pCDNA3(-)Pac and stop codons introduced early in the Vpu or Nef open reading frames by site-directed mutagenesis (based on the QuickChange system; Stratagene) using the following primers (mutagenized codons boxed):

Vpu forward: CATGTA<u>ATG</u>CAACCT[TGA]ATA[TAA]GCAATAGTAGCATTAG

Vpu reverse: CTAATGCTACTATTGC[TTA]TAT[TCA]AGGTTGCATTACATG

Nef forward: CTATAAG<u>ATG</u>GGTGGCAAG[TAGTGATAA]AGTAGTGTGATTGGATG

Nef reverse: CATCCAATCACACTACTTT[ATCACTACTT]GCCACCCATCTTATAG

Restriction fragments were sub-cloned back into pNL4-3-deltaE-EGFP and mutations verified by Sanger sequencing (Source BioScience), immunoblot of infected CEM-T4s for Vpu and Nef proteins, and functional analysis of infected CEM-T4s by flow cytometry for EGFP, CD4 and tetherin.

In addition, the following HIV-1 molecular clones were utilised for particular experiments:

pNL4-3 – replication-competent virus (WT, ΔVpu, ΔNef, ΔVpuΔNef and Vpu phosphodegron and transmembrane mutants), used for infectious virus release assays and validation of SNAT1 downregulation in primary human CD4+ T-cells and 293Ts expressing SNAT1-HA

pNL4-3 Vpu 2_87 – Env-deficient virus (WT, ΔVpu, and Vpu phosphodegron mutant) encoding Vpu RP2v16_2_87 (Pickering et al., 2014) on a pNL4-3 background, used for T-cell proliferation assays

Vectors for Transgene Expression

For lentiviral transgene expression in CEM-T4s and HeLas, codon-optimised NL4-3 Vpu was sub-cloned from pCR3.1-Vpu-HA (Vigan and Neil, 2010) into pHRSIN-PGK-puro (van den Boomen et al., 2014) with (experiments in HeLas) or without (experiments in CEM-T4s) the C-terminal HA tag. Codon-optimised, untagged NL4-3 Nef (based on pNL4-3, Genbank: AF324493) was synthesised (GenScript) and subcloned into pHRSIN-PGK-puro.

For lentiviral expression in primary human CD4+ T cells, the same untagged, codon-optimised transgenes were subcloned into pHRSIN-SE-P2A-SBP-ΔLNGFR-W, encoding Vpu or Nef co-translated as Vpu/Nef-P2A-SBP-ΔLNGFR for Antibody-Free Magnetic Cell Sorting using the SBP-ΔLNGFR cell surface affinity tag (Matheson et al., 2014). To generate codon-optimised Vpu encoding S52A and S56A point mutations (Vpu S52, 56A) pCR3.1-Vpu-HA was subjected to site-directed mutagenesis (based on a modified version of the QuickChange system (Zheng et al., 2004)) using the following primers (mutagenized codons boxed):

Forward: AGGACGCCGGCAACGAGGCCGAGGGCGAGGTGAGC

Reverse: CCCTCGGCCTCGTTGCCGGCGTCCTCGGCGCGCTC

Restriction fragments were sub-cloned into pHRSIN-SE-P2A-SBP-ΔLNGFR-W and mutations verified by Sanger sequencing (Source BioScience) and functional analysis of primary human CD4+ T cells transduced with Vpu- and Vpu S52, 56A-expressing lentiviruses using flow cytometry for LNGFR, CD4 and tetherin.

pCG-IRES-GFP vectors (mammalian expression vectors encoding Vpu or Nef variants as Vpu/Nef-IRES-GFP) have been previously described (Sauter et al., 2009). Sequence names identify HIV or SIV isolates from which Vpu variants are derived. Illustrative phylogenetic relationships were adapted from published phylogenetic trees based on Nef and Pol sequences (Kirchhoff, 2009; Schmokel et al., 2011; Sharp and Hahn, 2010; Takehisa et al., 2009). The SIVcpz lineage arose by recombination between SIVrcm and a common Vpu-encoding precursor of SIVgsn/mus/mon (SIVguenon). SIVden encodes a truncated Vpu protein and its phylogenetic relationship to the SIVgsn/mus/mon lineages is unclear (Dazza et al., 2005). HIV-1 and SIVgor viruses belong to a monophyletic cluster with SIVcpz viruses infecting the central *P. t. troglodytes* (*Ptt*) subspecies of chimpanzee (SIVcpz *Ptt*), with

SIVcpz viruses infecting the eastern *P. t. schweinfurthii* subspecies forming an outgroup (SIVcpz *Pts*). The control pCG-IRES-GFP vector (expressing GFP only) encodes a defective Nef with a mutation in the start codon and two premature stop codons (Sauter et al., 2009).

A SNAT1 cDNA clone was obtained from Open Biosystems in the pCMV-SPORT6 vector (IMAGE clone ID: 3871101; GenBank: BC010620.1; Entrez Gene ID: 81539 – *SLC38A1*). For lentiviral expression of full length C-terminally tagged SNAT1-FLAG, the following primer pairs were used for PCR amplification (FLAG sequence boxed):

Forward: GGATCCATCATGATGCATTTCAAAAGTGG

Reverse:
GGGCCCTCACTTATCGTCGTCATCCTTGTAATCTGCGGCCGCGTGGCCTTCGTCACTACTC

The resulting PCR products were ligated into pCR-Blunt-II TOPO, sequence-verified by Sanger sequencing (Source BioScience) and sub-cloned into pHRSIN-PGK-puro (van den Boomen et al., 2014) to generate pHRSIN-PGK-puro-SNAT1-FLAG.

For SNAT1 antibody production using GST-SNAT1 as an immunogen, the N-terminal 189 nucleotides of SNAT1 were amplified by PCR using the following primer pair:

Forward: GGATCCGCAGCTATGATGCATTTCAAAAGTGGAC

Reverse: GTCGACTCAACACTTCTTTTTTTCCAAATGGC

The resulting PCR product was ligated into pCR-Blunt II-TOPO, sequence verified by Sanger sequencing (Source BioScience) and sub-cloned into pGEX-4T-1 (a kind gift of Sally Gray) to create a single open reading frame encoding GST at the N-terminus fused with the N-terminal 63 amino acids of SNAT1 at the C-terminus (GST-SNAT1).

In addition, the following expression vectors were utilised for particular experiments:

pCR3.1 – mammalian expression vector encoding SERINC5, used for titration of HIV-1 infectious viral release

pCMS28 – pMigR1-based retroviral vector encoding Vpu-IRES-puro (Vpu WT, phosphodegron and transmembrane mutants), used for generation of stable Jurkat cell lines, or SNAT1-HA, used for generation of stable 293T cell lines expressing C-terminally tagged SNAT1-HA

pCSIG – pCS-based lentivector encoding Vpu RP2v16_2_87-IRES-GFP (Vpu WT, phosphodegron and transmembrane mutants), used for validation of SNAT1 downregulation in 293Ts expressing SNAT1-HA and T-cell proliferation assays

pQCXIH – pMSCV-based retroviral vector encoding human CD4-IRES-hygro (a kind gift from Professor Margaret Robinson), used for generation of stable 293T cell lines expressing CD4

Generation of the Vpu transmembrane mutants has been previously described (Vigan and Neil, 2010). As compared with NL4-3 Vpu phosphodegron (S52, 56A) and transmembrane (A14L, W22A) mutants, equivalent Vpu RP2v16_2_87 mutations are at residues S53, 57A and A15L, W23A, respectively.

Lentivectors for shRNA Expression

For lentiviral shRNA-mediated knockdown of SNAT1 in primary human CD4+ T cells, SNAT1-specific hairpins were cloned into pHRSIREN-S-SBP-ΔLNGFR-W (Matheson et al., 2014), encoding a U6-shRNA cassette and SFFV-SBP-ΔLNGFR for Antibody-Free Magnetic Cell Sorting using the SBP-ΔLNGFR cell surface affinity tag. Complementary shRNA-encoding oligonucleotides were designed using the Clontech on-line shRNA Sequence Designer, annealed, cloned into pHRSIREN using BamHI and EcoRI and sequence verified by Sanger sequencing (Source BioScience). The following (sense) targeting sequences were used:

SNAT1 shRNA KD 1: GGCAAACACTGGAATCCTA

SNAT1 shRNA KD 2: GATAGTTACCTTTGGCATA

SNAT1 shRNA KD 3: GAGAATCATGTGTACCTAA

Non-targeting control shRNA CTRL 1: GTTATAGGCTCGCAAAAGG

LacZ control shRNA CTRL 2: GTGACCAGCGAATACCTGT

The KD 1 sequence was selected using the Clontech on-line RNAi Target Sequence Selector. The KD 2 and KD 3 sequences were selected after deconvolution of a SNAT1-specific SMARTpool of four ON-TARGET*plus* siRNA oligonucleotides (Dharmacon). The CTRL 1 (control, scrambled) sequence (Matheson et al., 2014) and the CTRL 2 (control, targeting the bacterial *lacZ* gene) sequence (Qin et al., 2003) have been previously described. All sequences were searched against human RefSeq RNA using NCBI BLAST to confirm ≥4 mismatches with any contiguous off-target sequence.

KD 1 and CTRL 1 were used for all SNAT1 shRNA experiments except: KD 1, KD 2, CTRL 1 and CTRL 2 were used for Consumption and Release (CoRe) metabolomic analysis; and KD 3 and CTRL 1 were used for CellTrace Violet-based T-cell proliferation assays.

For generation of stable CEM-T4 cell lines, hairpins were cloned into pHRSIREN-PGK-puro, identical to pHRSIREN-S-SBP-ΔLNGFR-W but encoding PGK-puro for antibiotic selection (originally named pCSRQ and kindly provided by Professor Greg Towers) (Matheson et al., 2014; Schaller et al., 2011). For CellTrace Violet-based T-cell proliferation assays, hairpins were cloned into pHRSIREN-PGK-GFP, identical to pHRSIREN-S-SBP-ΔLNGFR-W and pHRSIREN-PGK-puro but encoding GFP for flow cytometric analysis.

siRNA Oligonucleotides

For transient siRNA-mediated knockdown of SNAT1, cells were transfected with a SNAT1-specific SMARTpool of four ON-TARGET*plus* siRNA oligonucleotides (Dharmacon). For β-TrCP and TSG101 knockdowns, siRNA oligonucleotides (Sigma) encoding the following (sense) targeting sequences were used:

β-TrCP siRNA: GTGGAATTTGTGGAACATC

TSG101 siRNA: CCUCCAGUCUUCUCUCGUC

The TSG101 sequence has been previously described (Hewitt et al., 2002). The β-TrCP sequence is fully conserved between β-TrCP1 and β-TrCP2 and effectively depletes both isoforms (Jin et al., 2003).

In all siRNA experiments, cells not subjected to knockdown were transfected with MISSION siRNA Universal Negative Control #2 (Sigma) at equivalent concentrations.

Transfections

FuGENE 6 (Promega; HIV-1 and lentivector production and general transfection), TransIT-293 (Mirus; general transfection) or polyethylenimine (Polysciences; HIV-1 and lentivector production) were used for plasmid DNA transfections in 293Ts. Oligofectamine (Invitrogen) was used for siRNA transfections in HeLas. siRNA oligonucleotides were used at a final concentration of 50nM and experiments were generally performed 48hrs after siRNA transfections. For TSG101 and β-TrCP knockdowns, a “two hit” approach was used, with an additional siRNA transfection after 24hrs (Hewitt et al., 2002).

Viral Stocks

VSVg-pseudotyped NL4-3-deltaE-EGFP HIV-1 viral stocks were generated by co-transfection of 293Ts with pNL4-3-deltaE-EGFP molecular clones and pMD.G at a ratio of 9:1 (µg) DNA and a DNA:FuGENE 6 ratio of 1µg:6µl. Media was changed the next day and viral supernatants harvested and filtered (0.45µm) at 48hrs prior to concentration with Lenti-X Concentrator (Clontech) and storage at -80°C.

VSVg-pseudotyped pHRSIN and pHRSIREN lentivector stocks were generated by co-transfection of 293Ts with lentivector, p8.91 and pMD.G at a ratio of 2:1:1 (µg) DNA and a DNA:FuGENE 6 ratio of 1ug:3µl. Viral supernatants were harvested, filtered, concentrated if required and stored as per NL4-3-deltaE-EGFP HIV-1 viral stocks.

VSVg-pseudotyped NL4-3 and NL4-3-Vpu 2_87 HIV-1 viral stocks were generated by co-transfection of 293Ts with pNL4-3 or pNL4-3-Vpu 2_87 molecular clones, respectively, and pCMV-VSV-G at a ratio of 5:1 (µg) DNA using polyethylenimine 1mg/ml (Polysciences). Media was changed the next day and viral supernatants harvested and filtered (0.45µm) at 48hrs then incubated at 37°C for 2hrs in the presence of 10µM $MgCl_2$ and 20U/ml DNAse (Roche) prior to concentration by ultracentrifugation through a 20% sucrose cushion and storage at -80°C.

VSVg-pseudotyped pCSIG lentivector stocks were generated by co-transfection of 293Ts with lentivector, pCRV1-HIV-1-Gag-Pol and pCMV-VSV-G at a ratio of 2:2:1 (µg) ratio using polyethylenimine 1mg/ml (Polysciences). Viral supernatants were harvested, filtered, DNAse-treated, concentrated and stored as per NL4-3 and NL4-3-Vpu 2_87 HIV-1 viral stocks.

VSVg-pseudotyped retroviral stocks were generated by co-transfection of 293Ts with pQCXIH or pCMS28 and plasmids encoding murine leukaemia virus (MLV) Gag-Pol and VSVg as previously described (Duncan et al., 2012; Randow, 2006).

Lentivector and NL4-3-deltaE-EGFP HIV-1 viral stocks were titred by infection/transduction of known numbers of relevant target cells under standard experimental conditions followed by flow cytometry at 48-72hrs to identify % infected/transduced cells. NL4-3 and NL43-Vpu 2_87 HIV-1 viral stocks were titred by infecting HeLa-TZM-bl indicator cells (AIDS Reagent Program, Division of AIDS, NIAD, NIH: Dr John C. Kappes) and counting focus-forming units by light microscopy following addition of X-Gal substrate at 48hrs.

All lentiviral and replication-competent HIV-1 viral work was performed under Biosafety Level 2 or 3 conditions, respectively.

Infections and Transductions

CEM-T4s were infected with concentrated HIV-1 viral stocks by spinoculation at 800g for 1-2hrs in a non-refrigerated benchtop centrifuge in complete media supplemented with 10mM HEPES. Primary human CD4+ T cells were transduced with concentrated HIV-1 or lentivector stocks using the same protocol 1 day after activation with CD3/CD28 Dynabeads. In experiments with reverse transcriptase inhibitors, cells were incubated with zidovudine (10uM) and efavirenz (100nM) (AIDS Reagent Program, Division of AIDS, NIAD, NIH) for 1hr prior to spinoculation, and inhibitors maintained at these concentrations during subsequent cell culture.

Stable Cell Lines

Stable CEM-T4 and HeLa cell lines were generated by transduction with pHRSIREN (control or SNAT1-specific shRNA expression) or pHRSIN (Vpu, Nef or GFP expression) lentivectors and selection with puromycin at 2µg/ml (CEM-T4) or 1µg/ml (HeLa). Stable Jurkat cell lines were generated by transduction with empty or Vpu-encoding pCMS28 retroviral vectors and selection with puromycin at 2µg/ml.

Stable SNAT1-FLAG and CD4-expressing 293T cell lines were generated by sequential transduction with pHRSIN-PGK-puro-SNAT1-FLAG and pQCXIH-CD4 and selection with puromycin at 1µg/ml and hygromycin at 200µg/ml. Transduced cells were screened by limiting dilution cloning and flow cytometry for high SNAT1-FLAG and CD4 cell surface expression. All experiments shown were conducted using clone S10.

Antibody-Free Magnetic Cell Sorting (AFMACS)

Antibody-Free Magnetic Cell Sorting of primary human CD4+ T cells using the streptavidin-binding SBP-ΔLNGFR affinity tag was carried out as previously described (Matheson et al., 2014). 3-5 days after primary activation and transduction with SBP-ΔLNGFR-encoding lentivectors, CD3/CD28 Dynabeads were removed using a DynaMag-2 magnet (Invitrogen). Cells were resuspended in Incubation Buffer (IB; PBS without calcium/magnesium, 2mM EDTA, 0.1% BSA) at 10^7 cells/ml and incubated with Dynabeads Biotin Binder (Invitrogen) at a bead-to-total cell ratio of 4:1 for 30mins at 4°C. Bead-bound cells expressing SBP-ΔLNGFR were selected using a DynaMag-2 magnet, washed to remove unbound non-transduced cells, then released from the beads by incubation in IB supplemented with 2mM biotin for 15mins at room temperature. Enrichment was assessed by flow cytometry for LNGFR pre- and post-selection. Cells were rested and then re-stimulated with CD3/CD28 Dynabeads 7-10 days after primary activation for use in downstream applications.

Tandem Mass Tag (TMT)-Based Plasma Membrane Profiling

Sample preparation

For the TMT-based HIV-1 infection timecourse, CEM-T4s were spinoculated with VSVg-pseudotyped NL4-3-deltaE-EGFP HIV-1 virus at a multiplicity of infection (MOI) of 10 in the presence or absence of reverse transcriptase inhibitors. Aliquots of cells were harvested sequentially, dead cells removed using the Miltenyi Dead Cell Removal kit, and plasma membrane enrichment carried out as previously described (Weekes et al., 2010; Weekes et al., 2012; Weekes et al., 2013; Weekes et al., 2014). All steps were performed on ice or at 4°C and washes were with ice-cold PBS with Ca/Mg pH 7.4 (Sigma) unless otherwise stated.

For aminooxy-biotinylation of plasma membrane proteins, $2x10^7$ viable cells per timepoint were washed and resuspended in a "one pot" aminooxy-biotinylation mix comprising 1mM sodium meta-periodate (Thermo Scientific), 100uM aminooxy-biotin (Biotium) and 10mM aniline (Sigma) in ice-cold PBS pH 6.7 and incubated for 30mins in the dark. After quenching with glycerol (1mM final concentration), cell pellets were washed and frozen at -80°C prior to downstream processing.

For affinity purification of aminooxy-biotinylated proteins, cell pellets were thawed on ice and lysed at 4°C for 30mins in TBS/1% Triton X-100 (high purity; Thermo Scientific) supplemented with Complete Protease Inhibitor Cocktail (without EDTA; Roche), 5mM iodoacetamide (Sigma) and 0.1mg/mL PMSF (Sigma). Nuclei and cell debris were removed by centrifugation and aminooxy-biotinylated plasma membrane proteins precipitated by incubation with High Capacity Streptavidin Agarose Resin (beaded agarose support; Thermo Scientific) at 4°C for at least 2hrs. Beads were transferred to Snap Cap spin columns (Thermo Scientific), reduced (100mM DTT) and alkylated (50mM iodoacetamide) at room temperature, and washed extensively using a vacuum manifold with (in total) >20 column volumes of TBS/1% Triton X-100 lysis buffer, PBS/0.5% SDS, TBS/6M urea, and, finally, H_2O. Beads were then transferred to small volume Screw Cap columns (Thermo Scientific) and incubated with 5ng/µl modified sequencing grade trypsin (Promega) in 100mM HEPES pH 8.5 at 37° for 6hrs. After digestion, peptide eluates were collected by centrifugation and stored at +4° prior to TMT labelling the next day.

For TMT labelling, Tandem Mass Tag 6-plex reagents (Thermo Scientific) were dissolved in anhydrous acetonitrile (0.8mg/40µl) and 10µl added to each peptide sample at a final acetonitrile concentration of 30% (v/v). Samples were labelled as follows: uninfected cells, 0hrs (TMT 126); 6hrs (TMT 127); 24hrs (TMT 128); 48hrs (TMT 129); 72hrs (TMT 130);

72hrs plus reverse transcriptase inhibitors (TMT 130). Following incubation at room temperature for 1 hr, reactions were quenched with hydroxylamine to a final concentration of 0.3% (v/v). Samples were mixed at a ratio of 1:1:1:1:1:1 and dried down to remove acetonitrile prior to off-line peptide fractionation.

Off-line High pH Reversed-Phase (HpRP) peptide fractionation

Tryptic peptides were subjected to HpRP-HPLC fractionation using a Dionex Ultimate 3000 powered by an ICS-3000 SP pump with an Agilent ZORBAX Extend-C18 column (4.6mm × 250mm, 5μm particle size). Mobile phases (H_20, 0.1% $NH_{4^\circ}H$ or MeCN, 0.1% $NH_{4^\circ}H$) were adjusted to pH 10.5 with the addition of formic acid and peptides were resolved using a linear 40min 0.1-40% MeCN gradient over 40mins at a 400μl/min flow rate and a column temperature of 15°C. Eluting peptides were collected in 15 s fractions. One hundred and twenty fractions covering the peptide-rich region were re-combined to give 10 samples for analysis. To preserve orthogonality, fractions were combined across the gradient, with each of the concatenated samples comprising 12 fractions which were 10 fractions apart. Re-combined fractions were dried down using an Eppendorf Concentrator and resuspended in 15μl MS solvent (3% MeCN, 0.1% TFA)

Mass spectrometry

Data for TMT labelled samples were generated using an Orbitrap Fusion Tribrid mass spectrometer (Thermo Scientific). Peptides were fractionated using an RSLCnano 3000 (Thermo Scientific) with solvent A comprising 0.1% formic acid and solvent B comprising 80% MeCN, 20% H_20, 0.1% formic acid. Peptides were loaded onto a 50cm Acclaim PepMap C18 column (Thermo Scientific) and eluted using a gradient rising from 10 to 25% solvent B by 90mins and 40% solvent B by 115mins at a flow rate of 250nl/min. MS data was acquired in the Orbitrap at 120,000fwhm between 400-1600 m/z. Spectra were acquired in profile with AGC 4.0e5. Ions with a charge state between 2+ and 6+ were isolated for fragmentation in top speed mode using the quadrupole with a 1.5 m/z isolation window. CID fragmentation was performed at 35% collision energy with fragments detected in the ion trap between 400-1200 m/z. AGC was set to 5.0e3 and MS2 spectra were acquired in centroid mode. TMT reporter ions were isolated for quantitation in MS3 using synchronous precursor selection. Ten fragment ions were selected for MS3 using HCD at 53% collision energy. Fragments were scanned in the Orbitrap at 60,000fwhm between 100-500 m/z with AGC set to 2.0e5. MS3 spectra were acquired in profile mode with injection parallelisation enabled.

Data processing and analysis

Raw MS files were processed using Proteome Discoverer 1.4.0.288 (Thermo Scientific). Data were searched against a concatenated human (UniProt, downloaded on 04/11/13) and HIV-1 (based on pNL4-3, Genbank: AF324493.2) database. VSVg (UniProt: P03522) and dEnv-EGFP-KDEL (inferred from the pNL4-3-deltaE-EGFP sequence) were substituted for Env. Precursor mass tolerance and fragment mass tolerance were set to 10ppm and 0.6Da, respectively, with a maximum of 2 missed tryptic cleavage sites. Percolator was used for post-processing of search results with a peptide false discovery rate of 0.01. Observed reporter ion intensities were adjusted for lot-specific isotopic impurities and missing quan values replaced with the minimum detected ion intensity. Protein abundances were calculated using unique peptides and normalised according to median protein ratios.

Gene Ontology Cellular Compartment (GOCC) terms were imported using Perseus 1.4.1.3 (downloaded from http://maxquant.org). To estimate the number of plasma membrane proteins quantitated, we counted proteins with GOCC terms "plasma membrane", "cell surface" and "extracellular" or with short, membrane-specific GOCC terms but no subcellular assignment (Weekes et al., 2010; Weekes et al., 2012; Weekes et al., 2013; Weekes et al., 2014). Proteins with no GOCC terms were excluded from the denominator.

For clustering according to profiles of temporal expression, proteins identified by >1 unique peptide and with a minimum fold change from 0 of >2 were analysed using the Short Time Series Expression Miner (STEM) 1.3.8 (downloaded from www.cs.cmu.edu/Xjernst/stem) with default settings (Ernst and Bar-Joseph, 2006; Ernst et al., 2005). Significantly enriched temporal profiles were identified using a permutation-based test as previously described (Ernst et al., 2005). Unadjusted p-values are shown, but the number of genes assigned to a particular profile was only considered significant it the Bonferroni-corrected p-value was <0.05.

For functional analysis of proteins in Cluster #35, enrichment of Gene Ontology Biological Process and Molecular Function terms against a background of all proteins quantitated was determined using the Database for Annotation, Visualization and Integrated Discovery (DAVID) 6.7 (accessed on 5/8/14 at http://david.abcc.ncifcrf.gov) with default settings (Huang da et al., 2009a, b). Annotation clusters with enrichment scores >1.3 (equivalent to a geometric means of all included enrichment p-values <0.05) and terms with Bonferroni-adjusted p-values (EASE scores) <0.05 were considered significant.

For the interactive spreadsheet of all TMT data, gene name aliases were added using GeneALaCart (accessed on 27/5/15 at https://genealacart.genecards.org) (Rebhan et al., 1997).

The complete HIV-1 infection timecourse mass spectrometry proteomics dataset has been deposited to the ProteomeXchange Consortium (Vizcaino et al., 2014) via the PRIDE Proteomics Identifications (Vizcaino et al., 2013) partner repository with the dataset identifier PXD002934 (accessible at http://proteomecentral.proteomexchange.org).

SIALC-Based Plasma Membrane Profiling

Sample preparation

For the SILAC-based HIV-1 infection timecourse, CEM-T4s pre-labelled with heavy lysine and arginine were spinoculated with VSVg-pseudotyped NL4-3-deltaE-EGFP HIV-1 virus at an MOI of 10, and cells pre-labelled with medium lysine and arginine were mock spinoculated without virus. Aliquots of HIV-infected (heavy) and mock (medium) cells were harvested sequentially at the indicated timepoints, dead cells removed using the Miltenyi Dead Cell Removal kit, and equal cell numbers mixed prior to plasma membrane enrichment as described below.

For the SILAC-based analysis of Vpu and Nef deletion virus infections, CEM-T4s pre-labelled with heavy lysine and arginine were spinoculated with VSVg-pseudotyped Nef-deficient NL4-3-deltaE-EGFP HIV-1 virus at an MOI of 10, cells pre-labelled with medium lysine and arginine were spinoculated with VSVg-pseudotyped Vpu-deficient NL4-3-deltaE-EGFP HIV-1 virus at an MOI of 10, and cells pre-labelled with light lysine and arginine were retained as uninfected controls. HIV-infected (heavy and medium) and control (light) cells were harvested 72hrs after infection, dead cells removed using the Miltenyi Dead Cell Removal kit, and equal numbers mixed prior to plasma membrane enrichment as described below.

For the SILAC-based analysis of Vpu and Nef single gene over-expression, CEM-T4s transduced with GFP were labelled with light lysine and arginine, cells transduced with Vpu were labelled with medium lysine and arginine, and cells transduced with Nef were labelled with heavy lysine and arginine. Dead cells were removed using the Miltenyi Dead Cell Removal kit, and equal numbers mixed prior to plasma membrane enrichment as described below.

Plasma membrane enrichment and off-line peptide fractionation were carried out as for the TMT-based HIV-1 infection timecourse, except that proteins were digested in Protein LoBind

microcentrifuge tubes (Eppendorf) with 10ng/µl modified sequencing grade trypsin (Promega) in 50mM ammonium bicarbonate at 37° overnight, and peptide eluates were not subjected to TMT labelling.

Mass spectrometry

Data for SILAC labelled samples were generated on a Q Exactive orbitrap mass spectrometer (Thermo Scientific). Peptides were fractionated using an RSLCnano 3000 (Thermo Scientific) with solvent A comprising 0.1% formic acid and solvent B comprising 80% MeCN, 20% H_20, 0.1% formic acid. Peptides were loaded onto a 50cm C18 EASYspray column (Thermo Scientific) and eluted using a gradient rising from 10% to 36% B by 75mins and 55% B by 100mins. MS data was acquired at 70,000fwhm between 400-1650 m/z with AGC of 1e6 and 250ms injection time. MS2 data was acquired at 17,500fwhm with AGC of 5e4, 200ms injection time and a loop count of 10. HCD fragmentation was performed at NCE of 28% and an underfill ratio of 20%.

Data processing and analysis

Raw MS files were processed using MaxQuant 1.3.0.5. Data were searched against a concatenated human (UniProt, downloaded 04/11/13) and HIV-1 (based on pNL4-3, Genbank: AF324493.2) database. VSVg (UniProt: P03522) and dEnv-EGFP-KDEL (inferred from the pNL4-3-deltaE-EGFP sequence) were substituted for Env. Fragment ion tolerance was set to 0.5Da with a maximum of 2 missed tryptic cleavage sites. Carbamidomethyl (C) was defined as a fixed modification, oxidation (M), acetylation (protein N-terminal) and deamidation (NQ) were selected as variable modifications. A reversed decoy database was used with the false discovery rate for both peptides and proteins set at 0.01. Peptide re-quantify was enabled and quantitation utilized razor and unique peptides. Normalized protein ratios are reported.

For comparisons of protein expression across TMT and SILAC experiments, datasets were combined according to UniProt accession numbers using Perseus 1.4.1.3. For validation of Cluster #35 downregulation in the SILAC-based HIV-1 infection timecourse, expression ratios of proteins in Cluster #35 were compared with expression ratios of all other proteins at each timepoint using 2-tailed 2-sample T-tests (assuming homoscedasticity) conducted using Excel.

Consumption and Release (CoRe) Metabolomics

Sample preparation

Primary human CD4+ T cells were activated with CD3/CD28 Dynabeads and transduced with pHRSIREN-S-SBP-ΔLNGFR-W lentivectors encoding control or SNAT1-specific shRNAs. Transduced cells were selected using Antibody-Free Magnetic Cells Sorting, rested and then re-stimulated using CD3/CD28 Dynabeads. After a further 24hrs, cells were resuspended at $5x10^5$ cells/ml in 20% conditioned media plus 10mM HEPES and supernatant samples obtained at baseline, 24 and 48hrs. For calculation of metabolite consumption/release, cell-free media samples were obtained at matched timepoints. Supernatants and media samples were stored at -80°C prior to analysis by mass spectrometry. The experiment was conducted in triplicate and cells enumerated at each timepoint using Dako CytoCount beads.

Metabolite profiling

Polar metabolites in media were profiled in both positive and negative ionization modes using two liquid chromatography tandem mass spectrometry (LC-MS) methods as described previously (Townsend et al., 2013). The methods were developed using authentic reference standards for each metabolite to determine chromatographic retention times and multiple reaction monitoring MS parameters, including declustering potentials and collision energies. Briefly, negative ionization mode data were acquired using an ACQUITY UPLC (Waters) coupled to a 5500 QTRAP triple quadrupole mass spectrometer (AB SCIEX). Media samples (30µL) were extracted using 120µl of 80% methanol containing 0.05ng/µl inosine-15N4, 0.05ng/µL thymine-d4, and 0.1ng/µl glycocholate-d4 as internal standards (Cambridge Isotope Laboratories). The samples were centrifuged (10mins, 9,000g, 4ºC) and the supernatants (10µl) were injected directly onto a 150mm x 2.0mm Luna NH2 column (Phenomenex, Torrance CA). The column was eluted at a flow rate of 400µl/min with initial conditions of 10% mobile phase A (20mM ammonium acetate and 20mM ammonium hydroxide (Sigma) in water (VWR)) and 90% mobile phase B (10mM ammonium hydroxide in 75:25 v/v acetonitrile/methanol (VWR)) followed by a 10min linear gradient to 100% mobile phase A. The ion spray voltage was -4.5kV and the source temperature was 500°C.

Positive ionization mode data were acquired using a 4000 QTRAP triple quadrupole mass spectrometer (AB SCIEX) coupled to a 1200 Series binary HPLC pump (Agilent) and an HTS PAL autosampler (Leap Technologies). Media samples (10µL) were extracted using nine volumes of 74.9:24.9:0.2 (v/v/v) acetonitrile/methanol/formic acid containing stable isotope-labelled internal standards (0.2ng/µl valine-d8, Isotec; and 0.2ng/µL phenylalanine-

d8 (Cambridge Isotope Laboratories)). The samples were centrifuged (10mins, 9,000g, 4ºC) and the supernatants (10µl) were injected onto a 150 x 2.1mm Atlantis HILIC column (Waters). The column was eluted isocratically at a flow rate of 250µl/min with 5% mobile phase A (10mM ammonium formate and 0.1% formic acid in water) for 1min followed by a linear gradient to 40% mobile phase B (acetonitrile with 0.1% formic acid) over 10mins. The ion spray voltage was 4.5kV and the source temperature was 450°C. Raw data were processed using MultiQuant 1.2 software (AB SCIEX) for automated peak integration. Metabolite peaks were manually reviewed for quality of integration and compared against reference standards to confirm identities.

Data analysis

For each supernatant sample, measured metabolite peak areas at 24 or 48hrs (S_{24} or S_{48}) were converted to per-cell consumption/release data ($CoRe_{24}$ or $CoRe_{48}$) by subtracting peak areas from cell-free media harvested at the same timepoint (M_{24} or M_{48}) and normalising according to average cell numbers. Changes in media volume over the 48 hr period were negligible. Cells numbers at baseline, 24 and 48hrs (n_0, n_{24} and n_{48}) were used to calculate average cell numbers from 0-24 and 0-48hrs as $(n_0 + n_{24})/2$ and $(n_0 + 2*n_{24} + n_{48})/4$, respectively. Therefore, for each metabolite: $CoRe_{24} = 2*(S_{24} - M_{24})/(n_0 + n_{24})$ and $CoRe_{48} = 4*(S_{48} - M_{48})/(n_0 + 2*n_{24} + n_{48})$. Metabolites with missing values were censored.

For multivariate comparison of metabolite profiles from control and SNAT1-depleted cells at 24 and 48hrs, CoRe data were autoscaled prior to Principal Component Analysis using MetaboAnalyst software (accessed at www.metaboanalyst.ca) (Xia et al., 2012; Xia et al., 2009; Xia et al., 2015). For univariate analysis of consumption/release of individual metabolites from control and SNAT1-depleted cells at 48hrs, 2-tailed 2-sample T-tests (assuming homoscedasticity) were conducted using Excel, with p-values corrected by the Bonferroni method. For graphical representation of individual metabolite consumption/release, CoRe data were scaled so that the maximum absolute value for each metabolite was equal to 1 (Jain et al., 2012).

3H-Alanine Uptake

3H-Alanine uptake was measured using an optimised method of silicone oil filtering centrifugation (Carr et al., 2010; Edinger and Thompson, 2002; Iiboshi et al., 1999; Pozueta-Romero et al., 1991; Werkheiser and Bartley, 1957). Primary human CD4+ T cells were activated with CD3/CD28 Dynabeads and transduced with pHRSIREN-S-SBP-ΔLNGFR-W or pHRSIN-SE-P2A-SBP-ΔLNGFR-W lentivectors encoding control or SNAT1-specific shRNAs, or Vpu or Vpu S52, 56A, respectively. Transduced cells were selected using

Antibody-Free Magnetic Cells Sorting, rested and then re-stimulated using CD3/CD28 Dynabeads. After a further 48-72hrs CD3/CD28 Dynabeads were removed using a DynaMag-2 magnet (Invitrogen) and cells enumerated using Dako CytoCount beads. Washed cells were then equilibrated in HBSS/2% dialysed FCS (Gibco) at $1x10^6$ cells/ml for 30-60mins at 37°C to deplete intracellular amino acids and reduce trans-inhibition (Iruloh et al., 2009; Iruloh et al., 2007). Starved cells were resuspended in Tyrode's buffer (NaCl 135mM, KCl 5mM, $CaCl_2$ 1.8mM, $MgCl_2$ 1mM, HEPES 10mM, glucose 5.6mM)/2% dialysed FCS at 2 $x10^6$ cells/ml and uptake at 37° initiated by addition of 3H-alanine (PerkinElmer) to a final concentration of 0.5mM (1µCi/timepoint). Aliquots of cells were harvested sequentially over 5mins. Each aliquot was transferred to the centre of the top layer of a pre-cooled 0.4ml microcentrifuge tube (Alpha Laboratories) containing: top layer 125µl PBS; middle layer 125µl silicone oil AR20/AR200 = 3/1 (Sigma); bottom layer 50µl 10% sucrose/H_2O). Tubes were spun immediately at 13,000g for 1min at 4°C, top and middle layers carefully aspirated using an extended length pipette tip (STARLAB) and cell pellets lysed for at least 30mins at 4°C by addition of 50µl 2% Triton/PBS. Lysates were combined with 4ml Ultima Gold MV (PerkinElmer) in Liquid Scintillation Counting vials (PerkinElmer) and counted on an LS 6500 scintillation counter (Beckman). Where specified, System A amino acid transport was inhibited with methylaminoisobutyric acid (MeAIB, 20mM; Abcam) (Mackenzie and Erickson, 2004; Tsitsiou et al., 2009). Control measurements with no cells routinely produced counts identical to background, suggesting highly efficient separation of cells from uptake buffer. Under the conditions used, 3H-Alanine uptake was found to be linear (corresponding to initial rate), Na-dependent and saturable with unlabelled alanine. Counts per minute (CPM) were plotted against time and linear regression lines with 95% confidence bands added using XLSTAT. Slopes (indicating rates of uptake) were compared using 2-tailed T-tests (assuming homoscedasticity) conducted using Excel.

Free Intracellular Amino Acids and Stable Isotopologue-Resolved Metabolomics

Sample preparation

Primary human CD4+ T-cells were expanded with CD3/CD28 Dynabeads, rested for 7-10 days, then re-stimulated using CD3/CD28 Dynabeads. After a further 48hrs CD3/CD28 Dynabeads were removed using a DynaMag-2 magnet (Invitrogen) and cells enumerated using Dako CytoCount beads. Washed cells were then resuspended at 37°C in RPMI supplemented with 10% dialysed FCS, glucose (5.6mM), glutamine (0.5mM) and alanine (concentrations as indicated). For stable isotope tracing, 13C6-glucose and 15N-alanine (both Cambridge Isotope Laboratories) were used. Where specified, System A amino acid transport was inhibited with methylaminoisobutyric acid (MeAIB, 20mM; Abcam) (Mackenzie

and Erickson, 2004; Tsitsiou et al., 2009). Aliquots of cells were harvested at the indicated timepoints, washed with ice-cold PBS, and snap frozen on dry ice. Free intracellular amino acids were extracted using dry ice-cold 50% methanol 30% acetonitrile and stored at -80°C prior to analysis by mass spectrometry. Cell size was determined by flow cytometry.

Mass spectrometry

LC-MS analysis was performed on a Q Exactive orbitrap mass spectrometer coupled to an Accela LC system (Thermo Scientific). The liquid chromatography system was fitted with either a Sequant Zic-HILIC column (column A, 150mm × 4.6mm, i.d. 3.5µm), or a Sequant Zic-pHilic (column B, 150mm × 2.1mm, i.d. 3.5µm) with guard columns (20mm × 2.1mm, i.d. 3.5µm) from HiChrom, Reading, UK. With column A, the mobile phase was composed by 0.1% aqueous formic acid (solvent A) and 0.1% formic acid in acetonitrile (solvent B). The flow rate was set at 300µl/min and the gradient was as follows: 0-3mins 80% B, 25mins 20% B, 26mins 80 % B, 36mins 80% B. For column B, the mobile phase was composed of 20mM ammonium carbonate and 0.1% ammonium hydroxide in water (solvent C), and acetonitrile (solvent D). The flow rate was set at 180µl/min with the following gradient: 0min 80% D, 28mins 20% D, 29mins 80% D, 45mins 80% D. The mass spectrometer was operated in full MS and polarity switching mode. Samples were randomised in order to avoid machine drifts. The acquired spectra were analysed using XCalibur Qual Browser and XCalibur Quan Browser softwares (Thermo Scientific). Mean metabolite abundance is expressed as fraction of maximum, with 95% confidence intervals shown for data obtained in triplicate.

SNAT1 Antibodies

Anti-SNAT1 (H60) rabbit polyclonal antibody (Santa Cruz Biotechnology) was used for Western blot and re-immunoprecipitation. Anti-SNAT1 (G63) rabbit polyclonal antibody (made in-house) was used for immunofluorescence microscopy and immunoprecipitation. To generate anti-SNAT1 (G63), glutathione S-transferase (GST)-SNAT1 fusion protein (incorporating the N-terminal 63 amino acids of SNAT1) was expressed in *E. coli* and purified from bacterial lysate by affinity chromatography using immobilised glutathione according to standard methods (GE, 2010). Unfused GST protein was also expressed and purified using empty pGEX-4T-1 vector. Fusion protein was detectable by immunoblotting with anti-SNAT1 (H60) and confirmed to run at approximately 33kDa by SDS-PAGE (compared with GST at approximately 26kDa) with no significant contaminating bands visualised by Coomassie staining. GST-SNAT1 was used to immunize 2 rabbits (Cambridge Research Biochemicals) and, after screening of crude sera by Western blot, serum from rabbit #5297 was selected for affinity purification. Serum was pre-sorbed against immobilised GST (to remove GST-specific antibodies) then purified by affinity

chromatography against immobilised GST-SNAT1 using the Pierce GST Orientation kit (Thermo Scientific) according to the manufacturer's instructions. Affinity-purified antibody was validated for detection of SNAT1 by immunofluorescence microscopy and immunoprecipitation using 293Ts over-expressing SNAT1 or depleted of SNAT1 by RNAi and CEM-T4s and primary human CD4+ T cells depleted of SNAT1 by RNAi. Aliquots were stored at -20°C in PBS/1% BSA/0.02% azide.

Other Antibodies

The following primary antibodies were used for flow cytometry (alphabetical order): anti-CD4-Brilliant Violet (clone OKT4; BioLegend); anti-CD4-Pacific Blue and anti-CD4-PE (clone RPA-T4; BD Biosciences); anti-CD25-PE (clone M-A251; BD Biosciences); anti-CD43-APC (clone CD43-10G7; BioLegend); anti-CD47-PerCP (clone CC2C6; BioLegend); anti-CD50 (clone TU41; BD Biosciences); anti-CD69-APC (clone FN50; BioLegend); anti-CD162-PE (clone KPL-1; BioLegend); anti-FLAG (rabbit polyclonal; Sigma); anti-LNGFR-APC and anti-LNGFR-PE (clone ME20.4; BioLegend); anti-NOTCH1-APC (clone MHN1-519; eBioscience); anti-p24-RD1 (clone KC57; Beckman Coulter); anti-tetherin (clone 3H4; Abnova) and anti-tetherin-APC (clone RS38E; BioLegend). As a functional assay for NCR3LG1 expression, recombinant human NKp30-Ig chimera (R&D Systems) was used in place of a primary antibody.

The following primary antibodies were used for immunoblots (alphabetical order): anti-α-tubulin (clone DM1A; Sigma); anti-ß-actin (clone AC-74; Sigma); anti-ß-TrCP (rabbit polyclonal H-300; Santa Cruz Biotechnology); anti-HA (clone HA.11/16B12; Covance); anti-HSP90 (rabbit polyclonal; Santa Cruz Biotechnology); anti-ubiquitin (clone VU-1; LifeSensors); anti-TSG101 (clone 4A10; GeneTex). Anti-Vpu and anti-p24 were obtained through the NIH AIDS Reagent Program, Division of AIDS, NIAID, NIH: Dr Klaus Strebel and Dr Bruce Chesebro, respectively (Maldarelli et al., 1993).

The following secondary antibodies were used: goat anti-mouse-AF647 and anti-rabbit-AF647 (flow cytometry; Molecular Probes); donkey anti-human-PE (flow cytometry; Jackson ImmunoResearch); goat anti-mouse-HRP and anti-rabbit-HRP (immunoblot, Jackson ImmunoResearch); donkey anti-goat-AF488 (immunofluorescence microscopy; Molecular Probes).

Flow Cytometry

Where necessary, CD3/CD28 Dynabeads were removed from primary human CD4+ T cells using a DynaMag-2 magnet (Invitrogen) prior to analysis. Typically $2x10^5$ washed cells were incubated for 30mins in 100µl PBS with the indicated fluorochrome-conjugated antibody or stained sequentially with primary and fluorochrome-conjugated secondary antibodies. All steps were performed on ice or at 4°C and stained cells were analysed immediately or fixed in PBS/1% paraformaldehyde. For NKp30-Ig staining, cells were first blocked for 30mins in PBS supplemented with 10% donkey serum (Sigma). For p24 staining, cells were first fixed and permeabilised using the BD Cytofix/Cytoperm method (BD Biosciences). Viability was assessed using forward and side scatter or, for CellTrace Violet-based T-cell proliferation assays, using the LIVE/DEAD Fixable Near-IR Dead Cell Stain kit (Molecular Probes). Where indicated, absolute cell numbers were determined using CytoCount beads (Dako) as a reference population. Cells were analysed using a BD FACSCalibur, FACSCanto II, LSRFortessa or ADP Cyan flow cytometer, and data processed using FlowJo software. For comparisons of fluorescence intensity, geomean fluorescence was used. For comparisons of cell size, mean intensity of forward-scattered light (FSC) was used.

Immunoblotting

Cells were typically lysed in TBS/1% Triton X-100 or PBS/1% SDS supplemented with 0.5mM phenylmethylsulfonyl fluoride (PMSF), 5mM iodoacetamide (IAA) and Complete Protease Inhibitor Cocktail (Roche) for 30mins on ice or at room temperature, respectively. For SDS lysis, Benzonase (Sigma) was included to reduce lysate viscosity. Post-nuclear supernatants were heated in Laemelli Loading Buffer for 25mins at 50°C, separated by SDS-PAGE and transferred to Immobilon-P membrane (Millipore). Membranes were blocked in PBS/5% non-fat dried milk (Marvel)/0.2% Tween and probed with the indicated primary antibody overnight at 4°C. Reactive bands were visualised using HRP-conjugated secondary antibodies and SuperSignal West Pico or Dura chemiluminescent substrates (Thermo Scientific). 8% Tris-glycine or 4-12% NuPAGE Bis-Tris gels were used to resolve SNAT1 and sample loading was by equivalent cell number (typically $1x10^5$/lane) or total protein (typically 10µg/lane; Pierce BCA Protein Assay kit; Thermo Scientific) depending on cell type. Where indicated, cells were pre-treated with MG132 (50µM for 4hrs; Cayman Chemical), lactacystin (20µM for 16hrs; Boston Biochem), concanamycin A (50nM for 16hrs; Calbiochem) or bafilomycin A1 (200nM for 16hrs; Calbiochem).

Immunoprecipitation

For co-immunoprecipitation of SNAT1 and Vpu-HA, 10^6 HeLas (transduced or transfected as indicated) were incubated with bafilomycin A (400nM for 4hrs; Calbiochem) prior to solubilisation in 1% Triton X-100. Lysates were pre-cleared with a combination of Protein A-Sepharose (Sigma) and IgG-Sepharose (GE Healthcare) and incubated for 1 hr at 4°C with anti-SNAT1 (G63)/Protein A-Sepharose (Sigma; for immunoprecipitation of SNAT1) or anti-HA coupled to agarose beads (Sigma EZview Red Anti-HA Affinity Gel; for immunoprecipitation of Vpu-HA). After washing in 1% Triton X-100, samples were eluted in SDS sample buffer, separated by SDS-PAGE, and immunoblotted as described.

For detection of ubiquitinated SNAT1, anti-SNAT1 (G63)-immunoprecipitated samples (as above) were washed in 1% Triton X-100 and denatured with 0.5% SDS before 50-fold dilution in 1% Triton X-100 and re-immunoprecipitation with anti-SNAT1 (H60). After further washing in 1% TritonX-100, samples were treated with Peptide-N-Glycosidase F (NEB) and separated by SDS-PAGE. Proteins were transferred to PVDF membranes and crosslinked with 0.5% glutaraldehyde/PBS for 20mins before probing with anti-ubiquitin (VU-1) and visualisation as described.

Immunofluorescence Microscopy

Where necessary, CD3/CD28 Dynabeads were removed from primary human CD4+ T cells using a DynaMag-2 magnet (Invitrogen) prior to analysis. Typically 2×10^5 cells were seeded in serum-free media for 30mins on 13mm coverslips pre-coated with 100µg/ml poly-D-lysine (Sigma). Cells were fixed and permeabilised for 5mins on ice with 100% MeOH pre-cooled to -20°C then blocked for 1 hr at room temperature in PBS/1% BSA supplemented with 5% donkey serum (Sigma) and Vivaglobin (160mg/ml human normal immunoglobulin; CSL Behring) 1/1000. Cells were stained sequentially for 1 hr at room temperature with anti-SNAT1 (G63) and donkey anti-goat-AF488 (Molecular Probes) in PBS/1% BSA and washed with PBS/0.1% Tween-20. For confocal microscopy, coverslips were mounted using ProLong Gold Antifade Mountant with DAPI (Invitrogen) before analysis using a Zeiss LSM510 or LSM710 confocal microscope. For Total Internal Reflection (TIRF) microscopy, coverslips were stained with DAPI 1/2000 in PBS and fixed in PBS/1% PFA for 15mins at room temperature before analysis with a Zeiss TIRF3 microscope. Representative images are shown.

Cell Proliferation

For titration of exogenous alanine in CEM-T4s and Jurkats, cells were seeded in a 96-well plate format in phenol red-free RPMI supplemented with 10% dialysed FCS, glutamine (2mM) and alanine (range of concentrations 0-0.3mM as indicated). Viable cell numbers were determined after 72hrs using an MTT (Thiazolyl Blue Tetrazolium Bromide) assay as previously described (Twentyman and Luscombe, 1987). After incubation with MTT (Sigma) 1mg/ml for 4hrs at 37°C, media was aspirated, formazan crystals solubilised in DMSO and optical density measured at 540nm.

For titration of exogenous alanine in primary human CD4+ T-cells, cells were stained with carboxyfluorescein succinimidyl ester (CFSE) and proliferation analysed according to standard methods. CFSE (Molecular Probes) was resuspended in DMSO at 5mM and stored at -20°C. Freshly isolated T-cells were incubated in 5μM CFSE/PBS/0.5% dialysed FCS at 10^7 cells/ml for 5mins at room temperature with continuous overhead rotation. Staining was quenched with 5 volumes of ice-cold complete medium and cells incubated on ice for 5mins. Washed cells were then resuspended in RPMI supplemented with 10% dialysed FCS, glucose (5.6mM), glutamine (0.5mM) and alanine (range of concentrations 0-0.3mM as indicated) before activation with CD3/CD28 Dynabeads. Cells were expanded with fresh media (containing matched concentrations of alanine) at 48hrs and CFSE fluorescence measured by flow cytometry after a further 72hrs.

For proliferation of primary human CD4+ T-cells transduced with GFP-expressing lentivectors, CellTrace Violet (Molecular Probes) was substituted for CFSE. Cells were typically activated with CD3/CD28 Dynabeads, transduced after 24hrs, and analysed by flow cytometry after a further 96hrs. CellTrace Violet fluorescence was evaluated in viable, GFP+ (transduced) cells. The same method was used to assess proliferation of primary human CD4+ T-cells infected with HIV-1, but CellTrace Violet fluorescence was evaluated in viable, p24+ (infected) cells. Mean % cells in each generation were compared using two-way repeated measures ANOVA conducted using Prism 5 (GraphPad Software).

For competition between exogenous alanine and MeAIB in primary human CD4+ T-cells, cells were seeded in a 96-well plate format in RPMI supplemented with 10% dialysed FCS, glucose (10.2mM), glutamine (0.6mM), alanine (range of concentrations 0-0.3mM as indicated) and MeAIB (range of concentrations 0-20mM as indicated) before activation with CD3/CD28 Dynabeads. Absolute cell numbers were determined by flow cytometry after 72hrs, using CytoCount beads (Dako) as a reference population.

Infectious Viral Release

For titration of SERINC5, 293Ts cells were co-transfected in a 24-well plate format with 500ng pNL4-3 HIV-1 molecular clones plus indicated concentrations of pCR3.1-SERINC5 using polyethylenimine 1mg/ml (Polysciences) and pCR3.1-GFP as a bulking plasmid. Culture supernatants were harvested after 48hrs. Infectious viral release was determined by infecting HeLa-TZM-bl indicator cells (AIDS Reagent Program, Division of AIDS, NIAD, NIH: Dr John C. Kappes) and measuring chemiluminescent β-galactosidase activity after 48hrs using the Tropix Galacto-Star system (Applied Biosystems) as previously described (Vigan and Neil, 2010). For SNAT1-HA over-expression, control and SNAT1-HA-expressing 293Ts were infected with VSVg-pseudotyped NL4-3 HIV-1 viruses at an MOI of 2. Culture supernatants were harvested after 48hrs, and infectious viral release determined by the same method.

SUPPLEMENTAL REFERENCES

Carr, E.L., Kelman, A., Wu, G.S., Gopaul, R., Senkevitch, E., Aghvanyan, A., Turay, A.M., and Frauwirth, K.A. (2010). Glutamine uptake and metabolism are coordinately regulated by ERK/MAPK during T lymphocyte activation. J Immunol *185*, 1037-1044.

Dazza, M.C., Ekwalanga, M., Nende, M., Shamamba, K.B., Bitshi, P., Paraskevis, D., and Saragosti, S. (2005). Characterization of a novel vpu-harboring simian immunodeficiency virus from a Dent's Mona monkey (Cercopithecus mona denti). J Virol *79*, 8560-8571.

Drakesmith, H., Chen, N., Ledermann, H., Screaton, G., Townsend, A., and Xu, X.N. (2005). HIV-1 Nef down-regulates the hemochromatosis protein HFE, manipulating cellular iron homeostasis. Proc Natl Acad Sci U S A *102*, 11017-11022.

Duncan, L.M., Timms, R.T., Zavodszky, E., Cano, F., Dougan, G., Randow, F., and Lehner, P.J. (2012). Fluorescence-based phenotypic selection allows forward genetic screens in haploid human cells. PLoS One *7*, e39651.

Edinger, A.L., and Thompson, C.B. (2002). Akt maintains cell size and survival by increasing mTOR-dependent nutrient uptake. Mol Biol Cell *13*, 2276-2288.

Ernst, J., and Bar-Joseph, Z. (2006). STEM: a tool for the analysis of short time series gene expression data. BMC Bioinformatics *7*, 191.

Ernst, J., Nau, G.J., and Bar-Joseph, Z. (2005). Clustering short time series gene expression data. Bioinformatics *21 Suppl 1*, i159-168.

Foley, G.E., Lazarus, H., Farber, S., Uzman, B.G., Boone, B.A., and McCarthy, R.E. (1965). Continuous Culture of Human Lymphoblasts from Peripheral Blood of a Child with Acute Leukemia. Cancer *18*, 522-529.

GE (2010). GST Gene Fusion System Handbook.

Hewitt, E.W., Duncan, L., Mufti, D., Baker, J., Stevenson, P.G., and Lehner, P.J. (2002). Ubiquitylation of MHC class I by the K3 viral protein signals internalization and TSG101-dependent degradation. EMBO J *21*, 2418-2429.

Huang da, W., Sherman, B.T., and Lempicki, R.A. (2009a). Bioinformatics enrichment tools: paths toward the comprehensive functional analysis of large gene lists. Nucleic Acids Res *37*, 1-13.

Huang da, W., Sherman, B.T., and Lempicki, R.A. (2009b). Systematic and integrative analysis of large gene lists using DAVID bioinformatics resources. Nat Protoc *4*, 44-57.

Iiboshi, Y., Papst, P.J., Kawasome, H., Hosoi, H., Abraham, R.T., Houghton, P.J., and Terada, N. (1999). Amino acid-dependent control of p70(s6k). Involvement of tRNA aminoacylation in the regulation. J Biol Chem *274*, 1092-1099.

Iruloh, C.G., D'Souza, S.W., Fergusson, W.D., Baker, P.N., Sibley, C.P., and Glazier, J.D. (2009). Amino acid transport systems beta and A in fetal T lymphocytes in intrauterine growth restriction and with tumor necrosis factor-alpha treatment. Pediatr Res *65*, 51-56.

Iruloh, C.G., D'Souza, S.W., Speake, P.F., Crocker, I., Fergusson, W., Baker, P.N., Sibley, C.P., and Glazier, J.D. (2007). Taurine transporter in fetal T lymphocytes and platelets: differential expression and functional activity. Am J Physiol Cell Physiol *292*, C332-341.

Jain, M., Nilsson, R., Sharma, S., Madhusudhan, N., Kitami, T., Souza, A.L., Kafri, R., Kirschner, M.W., Clish, C.B., and Mootha, V.K. (2012). Metabolite profiling identifies a key role for glycine in rapid cancer cell proliferation. Science *336*, 1040-1044.

Jin, J., Shirogane, T., Xu, L., Nalepa, G., Qin, J., Elledge, S.J., and Harper, J.W. (2003). SCFbeta-TRCP links Chk1 signaling to degradation of the Cdc25A protein phosphatase. Genes Dev *17*, 3062-3074.

Kirchhoff, F. (2009). Is the high virulence of HIV-1 an unfortunate coincidence of primate lentiviral evolution? Nat Rev Microbiol *7*, 467-476.

Koppensteiner, H., Hohne, K., Gondim, M.V., Gobert, F.X., Widder, M., Gundlach, S., Heigele, A., Kirchhoff, F., Winkler, M., Benaroch, P., *et al.* (2014). Lentiviral Nef suppresses iron uptake in a strain specific manner through inhibition of Transferrin endocytosis. Retrovirology *11*, 1.

Mackenzie, B., and Erickson, J.D. (2004). Sodium-coupled neutral amino acid (System N/A) transporters of the SLC38 gene family. Pflugers Arch *447*, 784-795.

Maldarelli, F., Chen, M.Y., Willey, R.L., and Strebel, K. (1993). Human immunodeficiency virus type 1 Vpu protein is an oligomeric type I integral membrane protein. J Virol *67*, 5056-5061.

Matheson, N.J., Peden, A.A., and Lehner, P.J. (2014). Antibody-free magnetic cell sorting of genetically modified primary human CD4+ T cells by one-step streptavidin affinity purification. PLoS One *9*, e111437.

Michel, N., Ganter, K., Venzke, S., Bitzegeio, J., Fackler, O.T., and Keppler, O.T. (2006). The Nef protein of human immunodeficiency virus is a broad-spectrum modulator of chemokine receptor cell surface levels that acts independently of classical motifs for receptor endocytosis and Galphai signaling. Mol Biol Cell *17*, 3578-3590.

Pickering, S., Hue, S., Kim, E.Y., Reddy, S., Wolinsky, S.M., and Neil, S.J. (2014). Preservation of tetherin and CD4 counter-activities in circulating Vpu alleles despite extensive sequence variation within HIV-1 infected individuals. PLoS Pathog *10*, e1003895.

Pozueta-Romero, J., Frehner, M., and Akazawa, T. (1991). Filtering centrifugation through two layers of silicone oil: a method for the kinetic analysis of rapid metabolite transport in organelles. Cell Struct Funct *16*, 357-363.

Qin, X.F., An, D.S., Chen, I.S., and Baltimore, D. (2003). Inhibiting HIV-1 infection in human T cells by lentiviral-mediated delivery of small interfering RNA against CCR5. Proc Natl Acad Sci U S A *100*, 183-188.

Ramirez, P.W., Famiglietti, M., Sowrirajan, B., DePaula-Silva, A.B., Rodesch, C., Barker, E., Bosque, A., and Planelles, V. (2014). Downmodulation of CCR7 by HIV-1 Vpu results in impaired migration and chemotactic signaling within CD4(+) T cells. Cell Rep *7*, 2019-2030.

Randow, F. (2006). Retroviral Transduction of DT40. In Reviews and Protocols in DT40 Research: Subcellular Biochemistry, J.-M. Buerstedde, ed. (Springer), pp. 383-386.

Rebhan, M., Chalifa-Caspi, V., Prilusky, J., and Lancet, D. (1997). GeneCards: integrating information about genes, proteins and diseases. Trends Genet *13*, 163.

Sauter, D., Schindler, M., Specht, A., Landford, W.N., Munch, J., Kim, K.A., Votteler, J., Schubert, U., Bibollet-Ruche, F., Keele, B.F.*, et al.* (2009). Tetherin-driven adaptation of Vpu and Nef function and the evolution of pandemic and nonpandemic HIV-1 strains. Cell Host Microbe *6*, 409-421.

Schaller, T., Ocwieja, K.E., Rasaiyaah, J., Price, A.J., Brady, T.L., Roth, S.L., Hue, S., Fletcher, A.J., Lee, K., KewalRamani, V.N.*, et al.* (2011). HIV-1 capsid-cyclophilin interactions determine nuclear import pathway, integration targeting and replication efficiency. PLoS Pathog *7*, e1002439.

Schmokel, J., Sauter, D., Schindler, M., Leendertz, F.H., Bailes, E., Dazza, M.C., Saragosti, S., Bibollet-Ruche, F., Peeters, M., Hahn, B.H.*, et al.* (2011). The presence of a vpu gene and the lack of Nef-mediated downmodulation of T cell receptor-CD3 are not always linked in primate lentiviruses. J Virol *85*, 742-752.

Sharp, P.M., and Hahn, B.H. (2010). The evolution of HIV-1 and the origin of AIDS. Philos Trans R Soc Lond B Biol Sci *365*, 2487-2494.

Takehisa, J., Kraus, M.H., Ayouba, A., Bailes, E., Van Heuverswyn, F., Decker, J.M., Li, Y., Rudicell, R.S., Learn, G.H., Neel, C.*, et al.* (2009). Origin and biology of simian immunodeficiency virus in wild-living western gorillas. J Virol *83*, 1635-1648.

Townsend, M.K., Clish, C.B., Kraft, P., Wu, C., Souza, A.L., Deik, A.A., Tworoger, S.S., and Wolpin, B.M. (2013). Reproducibility of metabolomic profiles among men and women in 2 large cohort studies. Clin Chem *59*, 1657-1667.

Tsitsiou, E., Sibley, C.P., D'Souza, S.W., Catanescu, O., Jacobsen, D.W., and Glazier, J.D. (2009). Homocysteine transport by systems L, A and y+L across the microvillous plasma membrane of human placenta. J Physiol *587*, 4001-4013.

Twentyman, P.R., and Luscombe, M. (1987). A study of some variables in a tetrazolium dye (MTT) based assay for cell growth and chemosensitivity. Br J Cancer *56*, 279-285.

van den Boomen, D.J., Timms, R.T., Grice, G.L., Stagg, H.R., Skodt, K., Dougan, G., Nathan, J.A., and Lehner, P.J. (2014). TMEM129 is a Derlin-1 associated ERAD E3 ligase

essential for virus-induced degradation of MHC-I. Proc Natl Acad Sci U S A *111*, 11425-11430.
Vigan, R., and Neil, S.J. (2010). Determinants of tetherin antagonism in the transmembrane domain of the human immunodeficiency virus type 1 Vpu protein. J Virol *84*, 12958-12970.
Vizcaino, J.A., Cote, R.G., Csordas, A., Dianes, J.A., Fabregat, A., Foster, J.M., Griss, J., Alpi, E., Birim, M., Contell, J.*, et al.* (2013). The PRoteomics IDEntifications (PRIDE) database and associated tools: status in 2013. Nucleic Acids Res *41*, D1063-1069.
Vizcaino, J.A., Deutsch, E.W., Wang, R., Csordas, A., Reisinger, F., Rios, D., Dianes, J.A., Sun, Z., Farrah, T., Bandeira, N.*, et al.* (2014). ProteomeXchange provides globally coordinated proteomics data submission and dissemination. Nat Biotechnol *32*, 223-226.
Weekes, M.P., Antrobus, R., Lill, J.R., Duncan, L.M., Hor, S., and Lehner, P.J. (2010). Comparative analysis of techniques to purify plasma membrane proteins. J Biomol Tech *21*, 108-115.
Weekes, M.P., Antrobus, R., Talbot, S., Hor, S., Simecek, N., Smith, D.L., Bloor, S., Randow, F., and Lehner, P.J. (2012). Proteomic plasma membrane profiling reveals an essential role for gp96 in the cell surface expression of LDLR family members, including the LDL receptor and LRP6. J Proteome Res *11*, 1475-1484.
Weekes, M.P., Tan, S.Y., Poole, E., Talbot, S., Antrobus, R., Smith, D.L., Montag, C., Gygi, S.P., Sinclair, J.H., and Lehner, P.J. (2013). Latency-associated degradation of the MRP1 drug transporter during latent human cytomegalovirus infection. Science *340*, 199-202.
Weekes, M.P., Tomasec, P., Huttlin, E.L., Fielding, C.A., Nusinow, D., Stanton, R.J., Wang, E.C., Aicheler, R., Murrell, I., Wilkinson, G.W.*, et al.* (2014). Quantitative temporal viromics: an approach to investigate host-pathogen interaction. Cell *157*, 1460-1472.
Werkheiser, W.C., and Bartley, W. (1957). The study of steady-state concentrations of internal solutes of mitochondria by rapid centrifugal transfer to a fixation medium. Biochem J *66*, 79-91.
Xia, J., Mandal, R., Sinelnikov, I.V., Broadhurst, D., and Wishart, D.S. (2012). MetaboAnalyst 2.0--a comprehensive server for metabolomic data analysis. Nucleic Acids Res *40*, W127-133.
Xia, J., Psychogios, N., Young, N., and Wishart, D.S. (2009). MetaboAnalyst: a web server for metabolomic data analysis and interpretation. Nucleic Acids Res *37*, W652-660.
Xia, J., Sinelnikov, I.V., Han, B., and Wishart, D.S. (2015). MetaboAnalyst 3.0-making metabolomics more meaningful. Nucleic Acids Res.
Zhang, H., Zhou, Y., Alcock, C., Kiefer, T., Monie, D., Siliciano, J., Li, Q., Pham, P., Cofrancesco, J., Persaud, D.*, et al.* (2004). Novel single-cell-level phenotypic assay for residual drug susceptibility and reduced replication capacity of drug-resistant human immunodeficiency virus type 1. J Virol *78*, 1718-1729.

Zheng, L., Baumann, U., and Reymond, J.L. (2004). An efficient one-step site-directed and site-saturation mutagenesis protocol. Nucleic Acids Res *32*, e115.
